# Transaminase-mediated synthesis of enantiopure drug-like 1-(3′,4′-disubstituted phenyl)propan-2-amines†

**DOI:** 10.1039/d0ra08134e

**Published:** 2020-11-10

**Authors:** Ágnes Lakó, Zsófia Molnár, Ricardo Mendonça, László Poppe

**Affiliations:** Department of Organic Chemistry and Technology, Budapest University of Technology and Economics Műegyetem rkp. 3 1111 Budapest Hungary poppe@mail.bme.hu +36-1-463-3299; Hovione Farmaciência, S.A., Campus do Lumiar Edifício R, Estrada do Paço do Lumiar 1649-038 Lisboa Portugal; Biocatalysis and Biotransformation Research Center, Faculty of Chemistry and Chemical Engineering, Babes-Bolyai University of Cluj-Napoca Arany János Str. 11 400028 Cluj-Napoca Romania

## Abstract

Transaminases (TAs) offer an environmentally and economically attractive method for the direct synthesis of pharmaceutically relevant disubstituted 1-phenylpropan-2-amine derivatives starting from prochiral ketones. In this work, we report the application of immobilised whole-cell biocatalysts with (*R*)-transaminase activity for the synthesis of novel disubstituted 1-phenylpropan-2-amines. After optimisation of the asymmetric synthesis, the (*R*)-enantiomers could be produced with 88–89% conversion and >99% ee, while the (*S*)-enantiomers could be selectively obtained as the unreacted fraction of the corresponding racemic amines in kinetic resolution with >48% conversion and >95% ee.

## Abstract 2

Immobilised whole-cell (*R*)-transaminases (TAs) enabled synthesis of either (*R*)- or (*S*)-enantiomers of drug-like amines from prochiral ketones or from racemic amines, respectively, in >95% ee.

## Graphical abstract

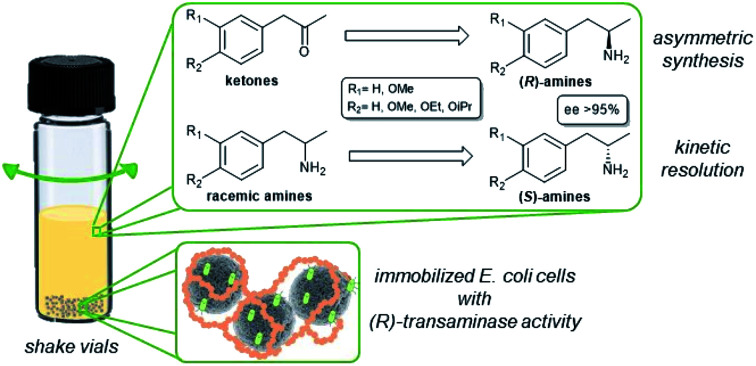

## Introduction

Chiral amines can be found as constituents in approximately 40% of all active pharmaceutical ingredients, but are also used as resolving agents for the separation of enantiomers,^1^ thus, there is a high demand for the synthesis of enantiomerically pure amines. For the synthesis of chiral amines, several solutions have been developed; however, these methods usually operate with chiral auxiliaries or metal complexes with chiral ligands and sometimes suffer from low enantioselectivity and low atom efficiency.^2^ Biocatalytic approaches *via* kinetic resolution (employing lipases^3^ monoamine oxidases^4^ or transaminases^5^), dynamic kinetic resolution with lipases,^6^ deracemisation with monoamine oxidases,^7^ or asymmetric synthesis (employing transaminases,^8^ amine dehydrogenases^9^ or imine reductases^10^) represent appealing alternatives for the synthesis of enantiopure amines.

**Fig. 1:**
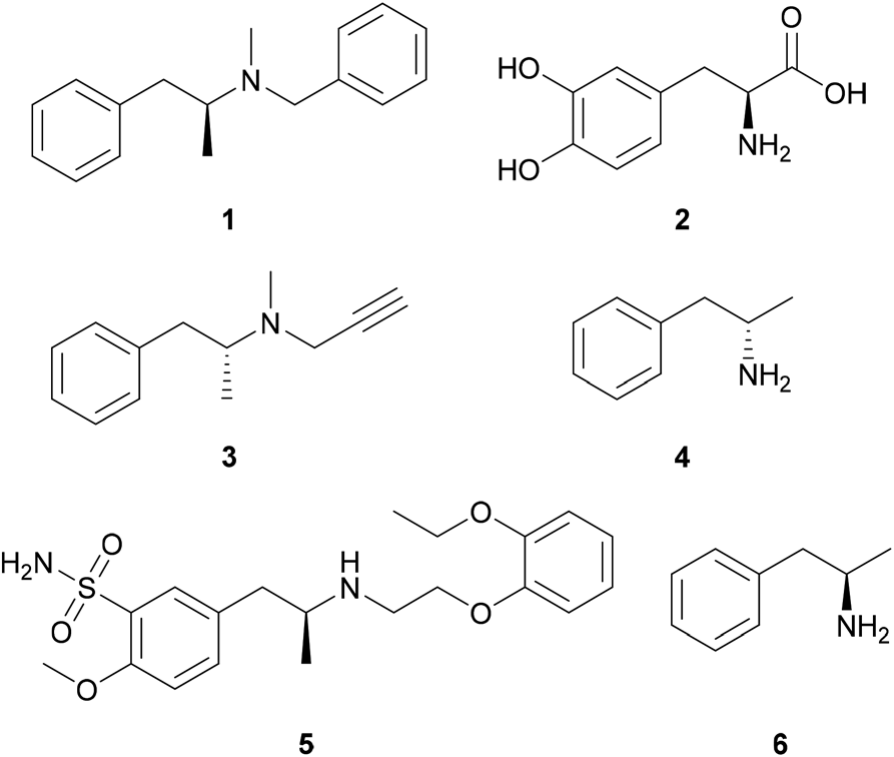
APIs containing the 1-phenylpropan-2-amine building block.

Commercial drugs (Fig. 1) containing both enantiomers of amphetamine and related compounds (1-arylpropan-2-amines) exist for the treatment of obesity (benzphetamine, 1),^11,12^ Parkinson's disease (l-DOPA,^13^2; selegiline,^14^3), narcolepsy and attention deficit hyperactivity disorder (dextroamphetamine, 4)^15,16^ and benign prostatic hyperplasia (tamsulosin, 5).^17^ Furthermore, l-amphetamine [6, (2*R*)-1-phenylpropan-2-amine] has been reported to improve cognitive function in multiple sclerosis patients.^18^

The synthesis of enantiopure (*S*)-1-phenylpropan-2-amine derivatives is more explored than of the (*R*)-amines; however, chemical and biocatalytic approaches both are reported for the enantioselective synthesis of l-amphetamine as well.

**Scheme 1:**
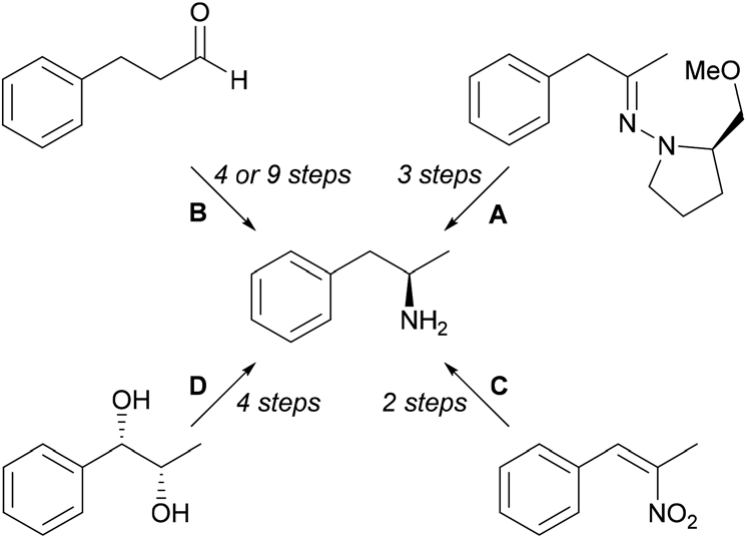
Chemical methods for the synthesis of (*R*)-1-phenylpropan-2-amine.

Some chemical methods (Scheme 1) for synthesis of l-amphetamine (6) include diastereoselective organocerium additions to (*S*)-1-amino-2-ethoxymethyl-pyrrolidine hydrazone derivatives followed by hydrogenolysis (A),^19^ proline-catalysed α-aminooxylation (9 steps) and α-amination (4 steps) strategies (B),^20^ hydrogenation of α,β-disubstituted nitroalkenes with rhodium and chiral phosphorus ligands (C),^21^ and Sharpless asymmetric dihydroxylation of olefins (D).^22^

**Scheme 2:**
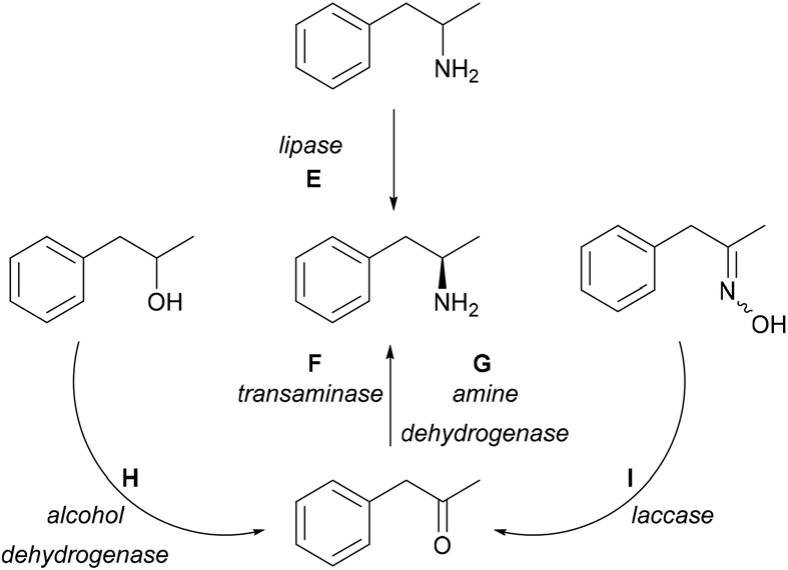
Biocatalytic syntheses of (*R*)-1-phenylpropan-2-amine.

Multiple biocatalytic strategies have been developed employing different (coupled) enzymes from various starting compounds (Scheme 2). Racemic 1-phenylpropan-2-amine can be enzymatically resolved *via* lipases (E);^23–26^ however, kinetic resolution only allows a maximum of 50% yield. The asymmetric reductive amination of carbonyl compounds with TAs (F) has been one of the main research areas of biocatalytic methods leading to chiral amines.^27,28^ TA-mediated amine syntheses have been widely reported as a one-enzyme system using a variety of amine donors without further transformation of the ketone co-product.^29–33^ Furthermore, there were reports on TAs coupled in cascades with pyruvate-reducing enzyme lactate dehydrogenase (LDH) and NADH-regenerating glucose dehydrogenase (GDH).^34,35^ Amine dehydrogenases (G, AmDH) can utilise ammonia for the synthesis of the desired amines by reductive amination of the prochiral ketone.^36^ Enzyme cascades further widen the possibilities. Starting from racemic alcohols, a five-enzyme system applying two enzymes for the synthesis [alcohol dehydrogenase (H, ADH), AmDH] and three enzymes [NADP-oxidase, catalase, and formate dehydrogenase (FDH)] for cofactor regeneration allowed the synthesis of the desired (*R*)-amines in near quantitative yields.^37^ An *in vitro* hydrogen-borrowing amination combining whole-cells with ADH and AmDH activity were applied in tandem operation,^38^ and was further developed to operate in *E. coli* cells co-expressing the two enzymes as well.^39^ This approach was also improved by employing a mutant AmDH,^36^ or by a sustainable immobilized system containing AmDH and GDH.^40^ Furthermore, ketoximes in a one-pot cascade consisting of laccases (I) and TAs (F) gave the desired compounds as well, although in this case, the starting materials were prepared from the ketone derivatives.^41^

Immobilisation of the biocatalysts can improve productivity, as the immobilised forms can have enhanced stability and can be reused in multiple catalytic cycles.^42–44^ Furthermore, whole-cell immobilisation combines the benefits of elimination of the high costs of enzyme purification and the possibility of reuse, thus representing a cost-efficient way of employing biocatalysts.^45^

Herein, we report the application of immobilized *E. coli* whole-cells overexpressing transaminases from *Arthrobacter* sp. (Ar*R*-TA and Ar*R*_m_-TA, natural and engineered, respectively) and *Aspergillus terreus* (At*R*-TA), the optimisation of the asymmetric synthesis of (*R*)-arylpropan-2-amines using 1-phenylpropan-2-one as model compound, and the kinetic resolution of the racemic amines providing access to the (*S*)-enantiomers.

## Results and discussion

**Scheme 3:**
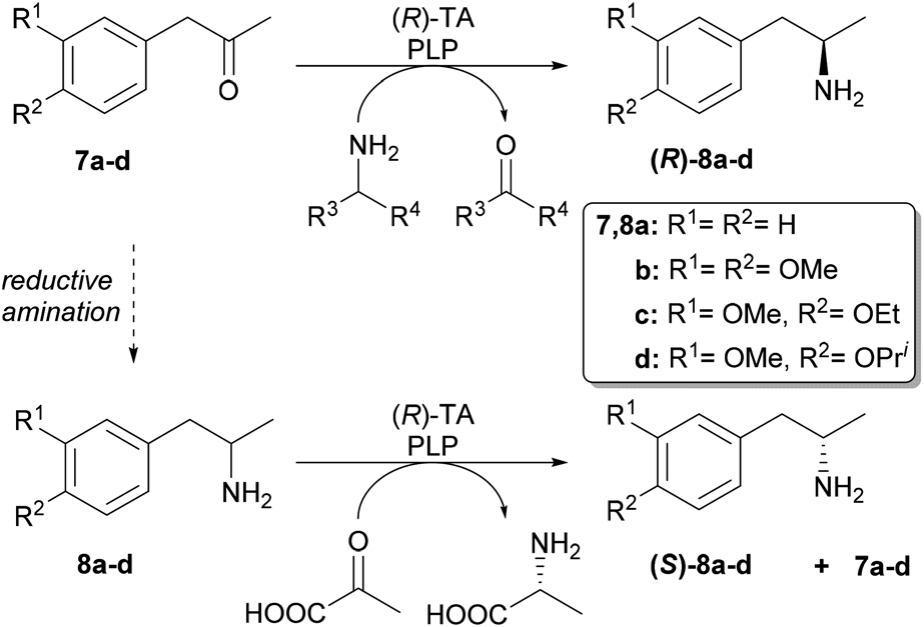
Transaminase-catalysed biotransformations leading to the (*R*)- and (*S*)-enantiomers of 1-arylpropan-2-amines 8a–d.

Our aim was to first optimise the asymmetric biocatalysis providing (*R*)-1-phenylpropan-2-amine (8a) from prochiral 1-phenylpropan-2-one (7a) with the aid of our sol–gel immobilised whole-cell TA biocatalysts.^46,47^ Additional goal was to extend the transamination with (*R*)-selective TAs to three further disubstituted derivatives (7b–d) as well (Scheme 3). Finally, to gain access to the (*S*)-enantiomers of the four amines by the (*R*)-selective TAs, we also investigated the kinetic resolution of all the racemic amines.

### Chemical synthesis of the prochiral 3,4-disubstituted 1-phenylpropan-2-ones (7b–d) and the corresponding racemic amines 8a–d

**Scheme 4:**
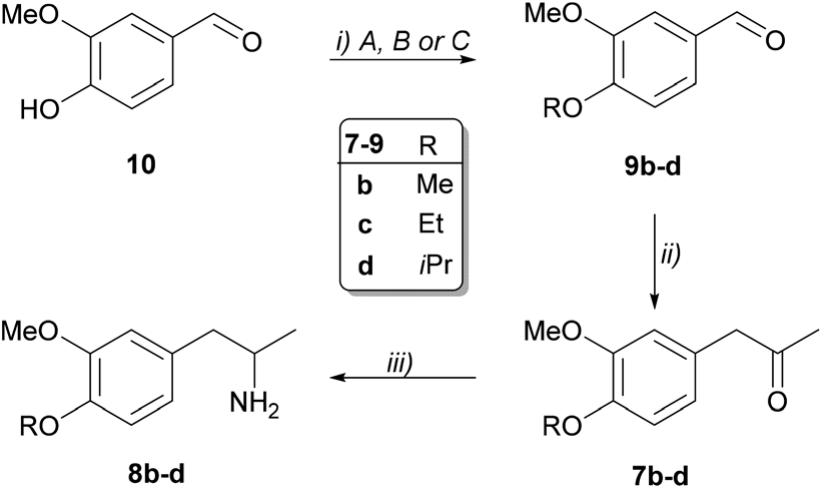
Synthesis of racemic disubstituted 1-phenylpropan-2-amines (8b–d). Reaction conditions: (i) A: 10 (20 mmol), K_2_CO_3_ (1 equiv.), Me_2_SO_4_ (2 equiv.) in 75 mL acetone, reflux; B: 10 (9.9 mmol), K_2_CO_3_ (1.5 equiv.), EtI (1.25 equiv.) in 15 mL DMF; C: 10 (6.6 mmol), K_2_CO_3_ (1.5 equiv.), iPrBr (1.5 equiv.) in 6.6 mL DMF, 80 °C; (ii) 9b–d (6 mmol), (±)-2-chloropropionic acid methyl ester (1.2 equiv.), NaOMe/MeOH (1.15 mmol, 25 wt%) in toluene (10 mL); 1N NaOH in toluene at 50 °C; 5N HCl in toluene, reflux; (iii) 7b–d (1 mmol), ammonium formate (10 equiv.), 10% Pd/C (0.04 equiv.) in 5 mL methanol.

Aromatic aldehydes 9b–d bearing substituents of various bulkiness were obtained from vanillin (10) by introducing three different alkyl groups (methyl, ethyl and isopropyl) with *O*-alkylation (Scheme 4). Darzens condensation from the *O*-alkylated aldehydes 9b–d resulted in the ketones 7b–d. Notably, efficient condensation required the use of freshly prepared sodium methoxide. Finally, Pd-catalysed transfer hydrogenation from the ketones 7a–d resulted in the racemic amines (8a–d) of sufficient purity enabling the further operations without purification.

### Reductive amination of 1-phenylpropan-2-one (7a) with (*R*)-selective whole-cell TA biocatalysts

The TA-mediated asymmetric synthesis of the (*R*)-amines was optimised in the biotransformations of the unsubstituted ketone 7a. Three immobilised TA-biocatalysts (Ar*R*-TA, Ar*R*_m_-TA, and At*R*-TA) were screened in the reductive amination of 7a with seven different amine donors including investigation of the effect of DMSO as cosolvent. To enhance productivity, substrate concentration was varied as well.

#### Amine donors

**Fig. 2:**
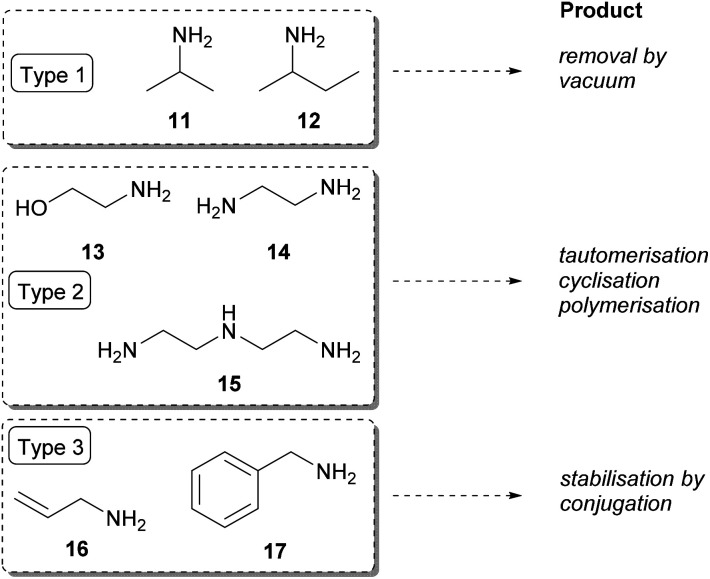
Various amine donors facilitating the displacement of the equilibrium of transamination.

In the asymmetric synthesis, for sufficiently high conversions the displacement of the reaction equilibrium to the product side is necessary.^48^ Since certain types of amine donors can contribute to the equilibrium displacement, the amine donors have a great impact on the yield. In the one-enzyme-based TA-systems, three types of amine donors have been considered (Fig. 2).

Type 1 amine donors are cheap and volatile; thus, these amines can be used in high excess to shift the reaction equilibrium towards product formation. Isopropylamine (11) and racemic *sec*-butylamine (12) and the corresponding ketones are low boiling-compounds that can be easily removed by vacuum.^8,49,50^ Although the pool of TAs which accept isopropylamine (11) as amine donor is limited, in case of acceptance industrial use of 11 is preferred compared to l-alanine, as the forming acetone can be easily removed.^51^ However, due to its unfavoured *K*_eq_, 11 has to be used in high excess and the forming acetone shows severe inhibitory effect. As an alternative, homologous *sec*-butylamine (12) was shown to be accepted in racemic form by TAs from *Arthrobacter* sp.^32^ and *Aspergillus terreus*^29^ as well. The more favourable *K*_eq_ with 12 allows use of the amine donor in smaller excess (*e.g.* 10 equiv. compared to 50 equiv.^52^), while the forming methyl ethyl ketone co-product is still volatile and doesn't show severe reaction inhibition.

The Type 2 amine donors [ethanolamine (13), ethylenediamine (14), diethylenetriamine (15)] can be considered as ‘*smart*’ co-substrates, where a coupled tautomerisation, dimerisation, or cyclisation of the carbonyl co-product to non-inhibiting co-products cause the equilibrium displacement.^53^

In case of Type 3 amine donors [allylamine (16) and benzylamine (17)], the forming carbonyl-compounds are energetically favoured due to conjugation of the carbonyl moiety to the C=C double bond or to the phenyl group.

Initially, the activity of all amine donors with the three TAs were screened in 10-fold excess (Table 1); however, using amines 13, 14 and 15 the conversion did not exceed 5% in any case (not shown in Table 1). The highest conversions could be achieved with all three (*R*)-TAs using *sec*-butylamine as amine donor, followed by isopropylamine being the second best. While Ar*R*-TA and At*R*-TA resulted in excellent ee in all cases (>99%), the highly mutated Ar*R*_m_-TA evolved for efficient transformation of a bulky substrate did not show perfect enantioselectivity.^8^

**Table 1:** Amine donor screening in transamination of 1-phenylpropan-2-one (7a) with three different immobilised transaminases^a^

Entry	Enzyme	Amine donor	Conversion [%]	ee [%]
1	Ar*R*-TA	Isopropylamine	27.8	>99 (*R*)
2		*sec*-Butylamine	83.0	>99 (*R*)
3		Allylamine	8.7	>99 (*R*)
4		Benzylamine	3.1	>99 (*R*)
5	Ar*R*_m_-TA	Isopropylamine	25.4	62.2 (*R*)
6		*sec*-Butylamine	51.6	82.4 (*R*)
7		Allylamine	14.4	91.5 (*R*)
8		Benzylamine	8.0	82.4 (*R*)
9	At*R*-TA	Isopropylamine	8.5	>99 (*R*)
10		*sec*-Butylamine	68.5	>99 (*R*)
11		Allylamine	0.8	>99 (*R*)
12		Benzylamine	2.8	>99 (*R*)

a Reaction conditions: immobilised whole-cell TA biocatalyst (20 mg), 7a (10 mM), amine donor (100 mM), PLP (1 mM), sodium phosphate buffer (100 mM, pH 7.5), DMSO (5 v/v%), 30 °C, 24 h.

**Fig. 3:**
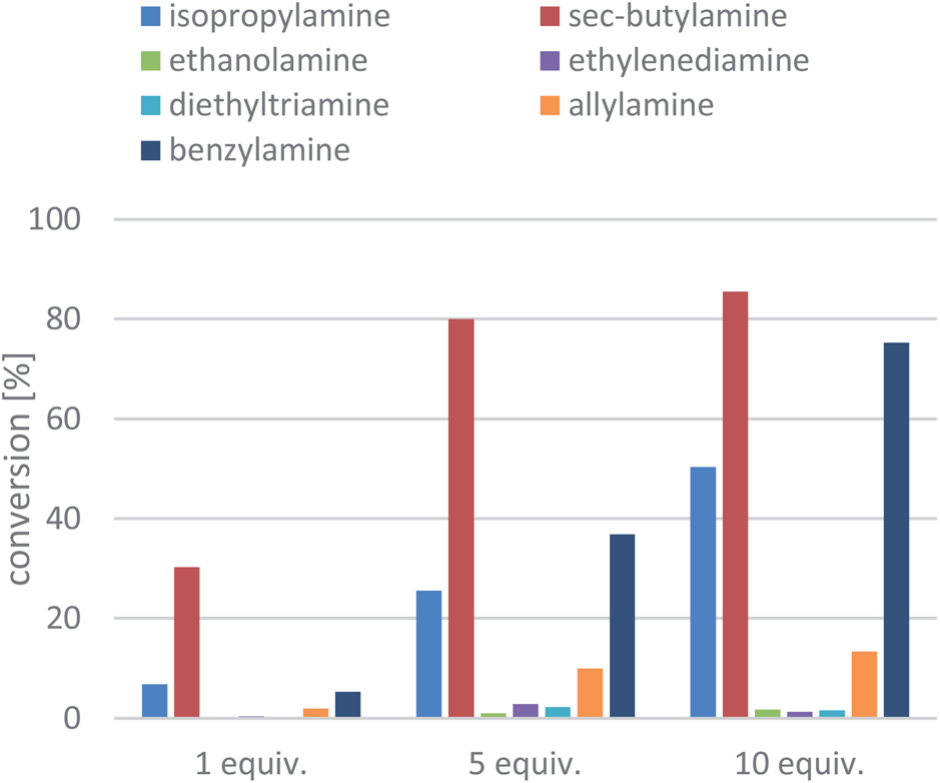
Screening the amine donors in Ar*R*-TA-mediated transamination of 1-phenylpropan-2-one (7a). Reaction conditions: immobilised whole-cell TA biocatalyst (20 mg), 7a (10 mM), amine donor (10–100 mM), PLP (1 mM), sodium phosphate buffer (100 mM, pH 7.5), DMSO (5 v/v%), 30 °C, 24 h.

Next, the amine donors in lower equivalencies were tested with the most active enzyme Ar*R*-TA, resulting in the transamination with excellent ee (>99%) but various conversions (Fig. 3). In this series of investigation, the reactions with 10-fold excess of amine donors (the usual minimal requirement of Type 1 amine donors, such as isopropylamine 11 or *sec*-butylamine 12) were compared to the reactions with no excess of amine donors (since smart co-substrates can drive the reaction to completion even in small excess), and to the ones with 5-fold excess of amine donors as well. Since in the lower than 10-fold equivalent amine donor cases the pH of the reaction remained within the operational range of the Ar*R*-TA, no pH correction was necessary. The results indicated that reactions with amine donors 13, 14, 15 and 16 were not sufficient for effective synthetic processes, thus they were not investigated any further. Unsurprisingly, with benzylamine 17 higher conversion could be achieved than with isopropylamine 11, reaching 75% conversion at 10-fold excess compared to that of 50% with isopropylamine. Because *sec*-butylamine 12 provided the highest conversion at any substrate : amine donor ratio (a reasonable 30.3% conversion could be achieved even with 1 equiv. of 12), the further optimisation was performed with 12 as amine donor. The significant difference between IPA (11) and SBA (12) can be rationalized by the more beneficial *K*_eq_, with 12 as amine donor due to the better affinity of the larger 12 to TAs as compared to 11.

#### Substrate concentration

**Fig. 4:**
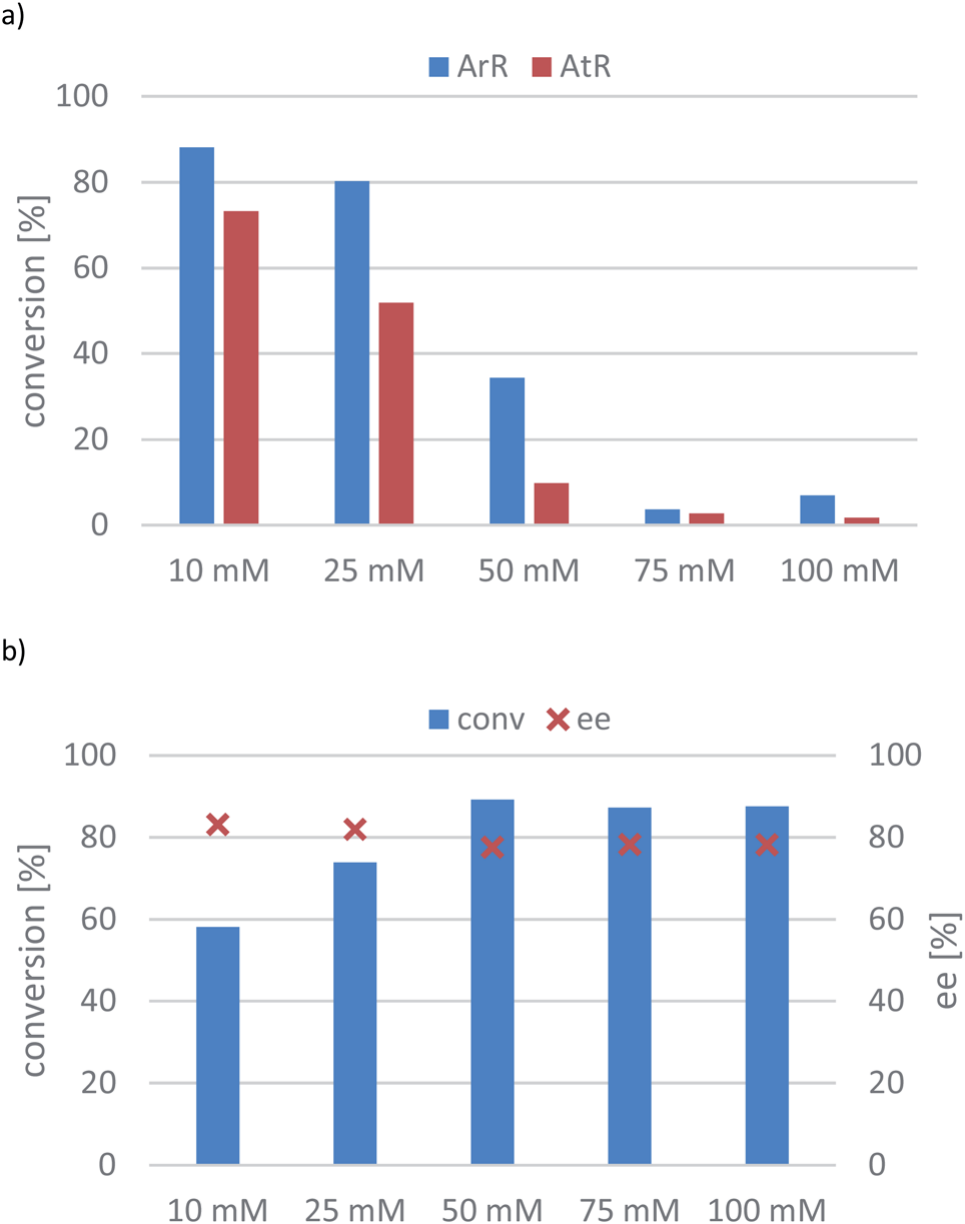
The effect of initial substrate concentration on the conversion of the transamination of 1-phenylpropan-2-one (7a) mediated by Ar*R*-TA or At*R*-TA (panel (a)); or Ar*R*_m_-TA (panel (b)). Reaction conditions: immobilised whole-cell TA biocatalyst (20 mg), 7a (10–100 mM), *sec*-butylamine 12 (0.1–1 M), PLP (1 mM), sodium phosphate buffer (100 mM, pH 7.5), DMSO (5 v/v%), 30 °C, 24 h.

In the next optimisation series, the substrate concentration was varied to explore the maximal concentration providing sufficiently high conversion (Fig. 4). In the reactions mediated by Ar*R*-TA and At*R*-TA, the conversion decreased gradually and significantly with increasing substrate concentration until <10% at 100 mM, while the ee remained excellent (>99%) (Fig. 4, panel (a)). In contrast, the evolved transaminase Ar*R*_m_-TA showed enhanced activity at higher concentrations reaching 89% conversion at 50 mM substrate concentration (Fig. 4, panel (b)).

Inspection of the substrate loading effect for 7a (Fig. 4) answered the question whether sol–gel immobilization could have protecting effect on the substrate/product inhibition of the transaminase-catalysed reaction. Because the conversions of the Ar*R*-TA- and At*R*-TA-catalysed reactions dropped significantly – especially with At*R*-TA – (Fig. 4a), it is apparent that sol–gel whole cell immobilization could not protect against substrate/product and this phenomena seems to be an intrinsic property of these two TAs (similarly to many other TAs). On the other hand, substrate inhibition was not a serious issue for Ar*R*_m_-TA (Fig. 4b), because this evolved TA retained its improved property of avoiding the substrate inhibition in sitagliptin intermediate production in the transamination of ketone 7a as well.

Although Ar*R*_m_-TA could operate with 88% conversion at 100 mM substrate concentration (10-fold increase compared to the initial tests), the enantiomeric excess remained around 80%. Since Ar*R*_m_-TA could not be used in highly enantiotope selective mode, the optimal substrate concentration was defined at 10 mM for the other two (*R*)-TAs.

#### Co-solvent

**Fig. 5:**
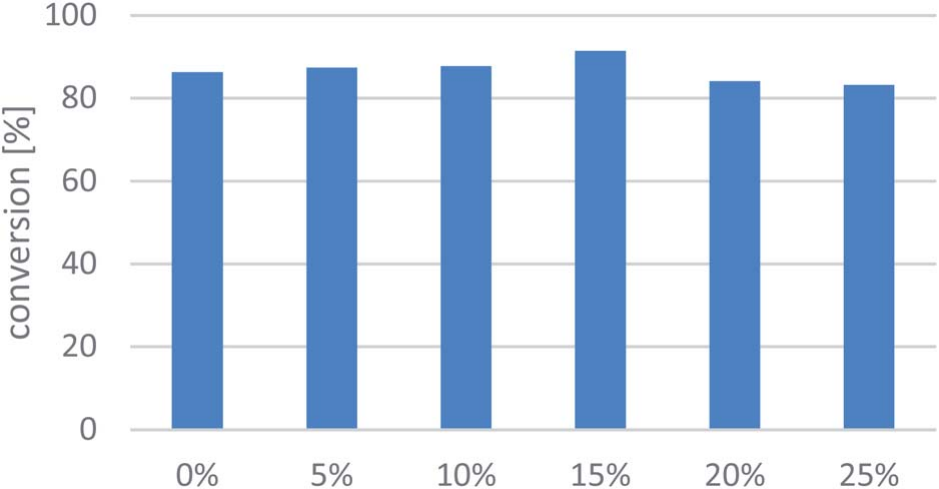
Cosolvent effect on conversion of the Ar*R*-TA-mediated transamination of 1-phenylpropan-2-one (7a). Reaction conditions: immobilised whole-cell TA biocatalyst (20 mg), 7a (10 mM), *sec*-butylamine 12 (100 mM), PLP (1 mM), sodium phosphate buffer (100 mM, pH 7.5), DMSO (0–25 v/v%), 30 °C, 24 h.

Native enzymes usually operate in aqueous media; however, examples have been developed for transamination in neat organic solvents,^54,55^ including transaminases from *Arthrobacter*.^56–58^ To overcome the limited substrate-solubility issue, 5% DMSO was applied even in the initial tests. However, to explore the further effect of DMSO on the immobilized Ar*R*-TA-mediated process, the amination of 7a was more thoroughly examined by adding various amounts of DMSO up to 25 v/v% to the buffer (Fig. 5). Although the highest conversion of transamination was observed at 15 v/v% DMSO content (*c* = 92%), conversions in the 0–25 v/v% DMSO content range exceeded 80% in all cases with excellent ee (>99%). In phosphate buffer without DMSO, the amine 7a resulted in formation of an emulsion, while increasing amounts of DMSO could harm the enzyme.^59^ Thus, the lowest 5 v/v% DMSO content providing sufficiently high conversion of 7a (*c* = 87.4%) was chosen as reaction medium for further experiments.

### Transamination of 1-phenylpropan-2-one derivatives 7a–d

The optimized conditions were applied for screening the synthesis of the disubstituted (*R*)-phenylpropan-2-amines (8b–d) mediated by the three selected immobilized whole-cell (*R*)-TA biocatalysts (Table 2). With Ar*R*-TA and At*R*-TA all the four 1-phenylpropan-2-ones 7a–d were transformed with similar efficiency (the conversions were 88–89% for Ar*R*-TA and 69–76% for At*R*-TA). Interestingly, although Ar*R*_m_-TA was evolved to transform a bulky substrate, this TA variant transformed the substituted derivatives 7b–d with significantly lower conversion (<36%) than the non-substituted ketone 7a (62%). Enantiotope selectivity of the Ar*R*_m_-TA-mediated amination of the smaller ketones 7a–c was moderate (ee = 84.9–88.5%). Expectedly, the highest enantiotope selectivity could be achieved in amination of 7d (ee = 92.7%) due to the increased bulkiness of this substrate.

**Table 2:** Transamination of 1-phenylacetone (7a) and the corresponding 3,4-disubstituted derivatives (7b–d) mediated by (*R*)-selective TAs^a^

Entry	Substrate	Enzyme	Conversion [%]	ee [%]
1	7a	Ar*R*-TA	88.9	>99 (*R*)
2		At*R*-TA	76.2	>99 (*R*)
3		Ar*R*_m_-TA	62.2	84.9 (*R*)
4	7b	Ar*R*-TA	88.0	>99 (*R*)
5		At*R*-TA	69.1	>99 (*R*)
6		Ar*R*_m_-TA	23.4	88.5 (*R*)
7	7c	Ar*R*-TA	88.5	>99 (*R*)
8		At*R*-TA	68.7	>99 (*R*)
9		Ar*R*_m_-TA	21.1	85.4 (*R*)
10	7d	Ar*R*-TA	89.0	>99 (*R*)
11		At*R*-TA	70.5	>99 (*R*)
12		Ar*R*_m_-TA	35.7	92.7 (*R*)

a Reaction conditions: immobilised whole-cell TA biocatalyst (20 mg), 7a–d (10 mM), *sec*-butylamine 12 (100 mM), PLP (1 mM), sodium phosphate buffer (100 mM, pH 7.5), DMSO (5 v/v%), 30 °C, 24 h.

To compare the efficiency of our TA biocatalysts, an estimated ∼0.1 recombinant TA-content per mg immobilized biocatalyst can be considered (for details, see Experimental section). However, comparison with previously reported methods can only made in a qualitative and not quantitative way. The various methods used the TA biocatalysts in different forms and applied various amine donors as well.

For the transamination of 7a, commercially available purified TAs,^30,60^ lyophilized whole cells^41^ or cell-free extracts (CFEs) directly^29^ were applied as TA-biocatalysts. In case of lyophilized whole cells, 250% w/w TA-biocatalyst was used for transforming 17 mM of 7a with 1 M IPA (∼60 equiv.) and 1 mM PLP for 24 h at 30 °C.^41^ TA as CFE (∼2 mg protein) was also employed for conversion of 7a (10 mM) in presence of 5–10 equiv. amine donor (l-alanine, IPA, SBA, methylbenzylamine) and 0.1 mM PLP for 24 h at 30 °C.^29^

The early reports on transamination of molecule 7b applied cells from fermentation broth and defined the quantity of catalyst only as whole cells harvested from 100 mL culture broth.^31,32,61^ Only one report described the production of (*R*)-8b using commercially available TAs,^60^ using 2 mg of purified (*R*)-selective TA in to transform 7b (20 mM) with IPA (11, 50 equiv.) and PLP (1 mM) for 24 h at 30 °C in 91% conversion. When the enzyme load was increased to 1 : 1 w/w TA : substrate ratio, the amine donor equivalent could be lowered to 16.

Thus, in this work the TA-mediated reactions with already investigated substrates 7a,b proceeded with similar results as previously disclosed, and data on novel substrates 7c,d extended the applicability of the immobilized whole-cell TA form.

### Kinetic resolution of the racemic amines 8a–d

**Fig. 6:**
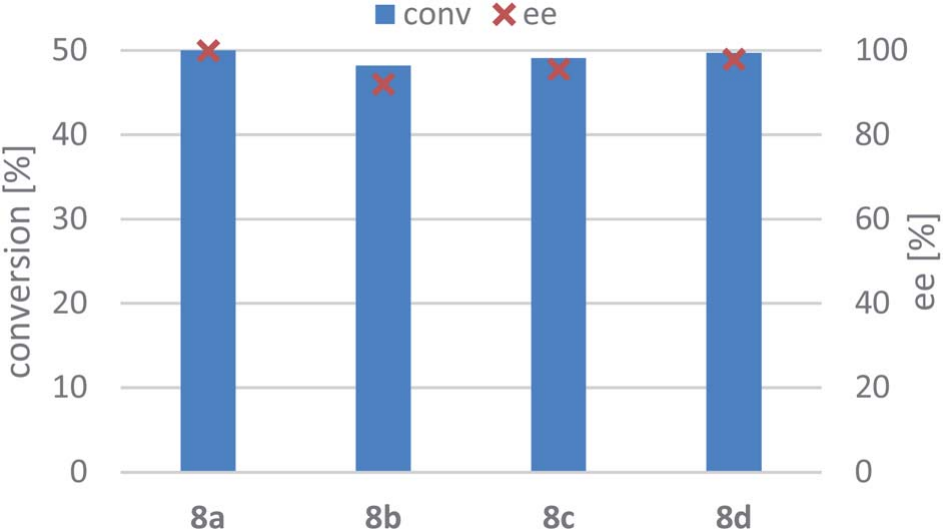
At*R*-TA-mediated kinetic resolution of the racemic amines 8a–d. Reaction conditions: immobilised whole-cell TA biocatalyst (20 mg), 8a–d (10 mM), sodium pyruvate (5 mM), PLP (1 mM), sodium phosphate buffer (100 mM, pH 7.5), DMSO (5 v/v%), 30 °C, 24 h.

Due to thermodynamic equilibrium reasons in the TA-mediated processes, kinetic resolution (KR) of racemic amines is more preferred to perform than asymmetric synthesis, however, at maximum conversion of the racemic substrate only 50% yield of the unconsumed enantiomer that can be achieved.^62^ Because in our preliminary screens none of the (*S*)-selective TAs in our hands (Ar*S*-TA, Vf*S*-TA, Cv*S*-TA_m_)^47^ showed sufficient activity in the asymmetric synthesis of (*S*)-8a–d, these (*S*)-amines were accessed by the (*R*)-selective TAs also, using KR of the corresponding racemic amines 8a–d (Scheme 3, Fig. 6). In all cases, At*R*-TA showed the highest activity and resulted in almost full conversions (>48%). Three substrates (8a, 8c and 8d) could be resolved efficiently with high selectivity (ee > 95%, *c* > 49%) while KR of 8b proceeded with lower conversion (*c* = 48.2%) reaching only 91% ee within 24 h.

## Experimental

### Methods and materials

#### Materials

If not stated otherwise, all chemicals and starting materials were purchased from Sigma-Aldrich (St. Louis, MO, USA), Fluka (Milwaukee, WI, USA) or Alfa Aesar Europe (Karlsruhe, Germany).

#### Biocatalysts

The immobilized whole-cell TA biocatalysts (At*R*-TA, Ar*R*-TA, and Ar*R*_m_-TA) were prepared as described in our previous work.^47^ Briefly, production of At*R*-TA, Ar*R*-TA, and ArR_m_-TA was achieved in *E. coli* BL21(DE3) containing the recombinant pET21a plasmid with the gene of the given TA.^63^ The *E. coli* cells containing the overexpressed TAs and hollow silica microspheres as supporting agent were immobilized by an improved sol–gel process.^47^

The TAs used had high expression level (∼40% of all proteins in the cell, with expressions plasmids using T7 promoter, even 50% recombinant protein content could be achieved).^64^ On average, wet *E. coli* cell mass contains 200–320 mg mL^−1^ protein,^65^ and the density of the cells is ∼1.1 g cm^−3^.^66^ Furthermore, it is known from our previous studies that ∼0.9 g of such supported whole-cell TA biocatalyst can be produced from 1 g of wet *E. coli* cells.^46,47^ Thus, the estimated recombinant TA content of 1 g immobilized *E. coli* cells is ∼90–140 mg. Consequently, our supported whole-cell TA-biocatalysts contained approximately 8–13% w/w recombinant TA (as a part of the ∼20–33% w/w total protein content).

#### Analytical and separation methods

NMR spectra were recorded in the indicated deuterated solvents on a Bruker DRX-300 or a Bruker DRX-500 spectrometer operating at 300 MHz and 500 MHz for ^1^H, 75 MHz and 126 MHz; for ^13^C. NMR signals are given in ppm on the *δ* scale. Infrared spectra were recorded on a Bruker ALPHA FT-IR spectrometer (in ATR mode) and wavenumbers (*ν*) of bands are listed in cm^−1^. High-resolution mass spectra were recorded on an ABSCIEX TripleTOF® 6600 System. TLC was carried out on pre-coated TLC ALUGRAM® Xtra SIL G/UV_254_ sheets (Macherey–Nagel). Spots were visualized under UV light (254 nm) column chromatography was carried out with Gerduran® Si 60 (Merck) silica gel. Gas chromatographic (GC) analyses were performed with an Agilent 4890 gas chromatograph equipped with FID detector using H_2_ carrier gas (injector: 250 °C, detector: 250 °C, head pressure: 12 psi, split ratio: 50 : 1) and Hydrodex β-6TBDM column [25 m × 0.25 mm × 0.25 μm film with heptakis-(2,3-di-*O*-methyl-6-*O-t*-butyldimethylsilyl)-β-cyclodextrine; Macherey & Nagel].

#### Calculations

Conversion (*c*) and enantiomeric excess values (ee) were determined by GC measurements with base-line separations of the peaks for the enantiomers of racemic amines 8a–d (see Section 1.2 in ESI†). The enantiomeric excess of the enantioenriched forms of amines 8a–d was calculated by the following formula:
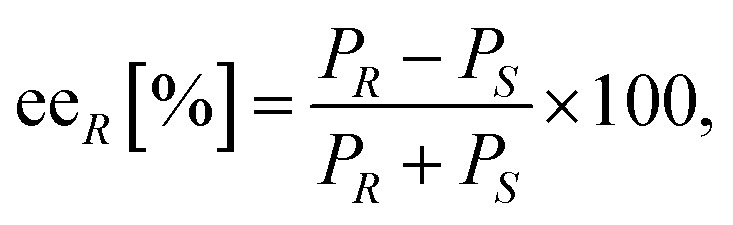
where ee_*R*_ is the enantiomeric excess of the (*R*)-amine, *P*_*R*_ and *P*_*S*_ indicate the peak area of the corresponding enantiomer of the investigated amine 8a–d.

The conversion of the racemic amines 8a–d in the highly selective kinetic resolution (*c*_kr_) was calculated by the following equation:
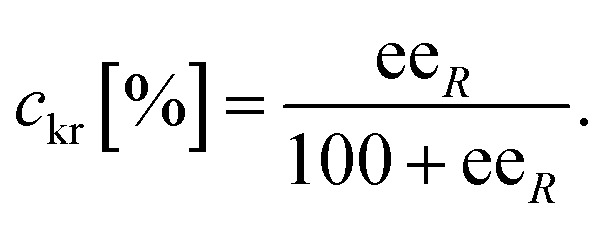


The molar response factor (*f*) of the amine 7a–d related to the corresponding starting ketone 8a–d was also determined from KR data as follows:
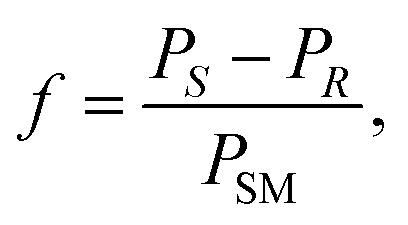
where *P*_SM_ indicates the peak area of the starting material.

The conversion of the ketones 7a–d in the asymmetric synthesis (*c*_as_) was calculated using the response factor *f* as follows:
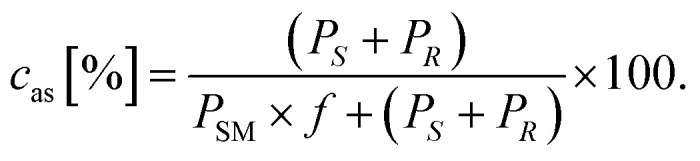


### Synthesis of 3,4-disubstituted benzaldehydes (9b–d)

#### 3,4-Dimethoxybenzaldehyde (9b)

To a solution of vanillin 10 (20 mmol) in acetone (75 mL) were added potassium carbonate (20 mmol, 1 equiv.) and dimethyl sulphate (40 mmol, 2 equiv.) at room temperature. The resulting suspension was refluxed for 90 min and allowed to cool to room temperature. The excess of dimethyl sulphate was neutralized by dropwise addition of triethylamine and the reaction mixture was concentrated. The residue was dissolved in dichloromethane (40 mL), washed with a 10% w/w sodium hydroxide solution (20 mL), and then with water (2 × 15 mL). The organic phase was dried over sodium sulphate, filtered, and evaporated. The crude residue was purified by column chromatography (eluent: hexane/ethyl acetate 10 : 4, TLC: *R*_f_ = 0.4) to give aldehyde 9b (2.42 g, 73%). White crystals, mp: 42–43 °C. ^1^H-NMR (500 MHz, CDCl_3_) 9.85 (s, 1H), 7.46 (dd, *J* = 8.2, 1.9 Hz, 1H), 7.41 (d, *J* = 2.0 Hz, 1H), 6.98 (d, *J* = 8.1 Hz, 1H), 3.97 (s, 4H), 3.94 (s, 3H); IR (cm^−1^) 2844, 1685, 1587, 1514, 1271, 1138. Physical and spectral data are in accordance with ref. 67.

#### 4-Ethoxy-3-methoxybenzaldehyde (9c)

Vanillin 10 (9.9 mmol) and potassium carbonate (14.8 mmol, 1.5 equiv.) were suspended in DMF (15 mL). Ethyl iodide (12.4 mmol, 1.25 equiv.) was added in one portion. The mixture was stirred at room temperature for 3 h, then poured onto water (40 mL) and extracted with ethyl acetate (3 × 15 mL). The combined organic layers were washed alternately with water and brine (3 × 10 mL, each) and dried over anhydrous sodium sulphate, filtered, and evaporated to give aldehyde 9c (1.51 g, 85%). White crystals, mp: 62–63. ^1^H-NMR (500 MHz, CDCl_3_) 9.87 (s, 1H), 7.46 (dd, *J* = 8.1, 1.9 Hz, 1H), 7.43 (d, *J* = 1.8 Hz, 1H), 6.99 (d, *J* = 8.1 Hz, 1H), 4.22 (q, *J* = 7.0 Hz, 2H), 3.96 (s, 3H), 1.54 (t, *J* = 7.0 Hz, 3H); IR (cm^−1^) 1684, 1585, 1509, 1266, 1137. Physical and spectral data are in accordance with ref. 68.

#### 4-Isopropoxy-3-methoxybenzaldehyde (9d)

To a solution of vanillin 10 (6.6 mmol) and potassium carbonate (9.9 mmol, 1.5 equiv.) in DMF (6.6 mL) was added isopropyl bromide (9.9 mmol, 1.5 equiv.) and the solution was stirred at 80 °C for 2 h. After cooling to room temperature, the solution was diluted with water (20 mL), extracted with diethyl ether (3 × 20 mL), washed with brine (20 mL), dried over sodium sulphate and evaporated to give aldehyde 9d (1.26 g, 99%). Colorless oil. ^1^H-NMR (500 MHz, CDCl_3_) 9.84 (s, 1H), 7.43 (dd, *J* = 8.1, 1.9 Hz, 1H), 7.41 (d, *J* = 1.9 Hz, 1H), 6.98 (d, *J* = 8.2 Hz, 1H), 4.69 (m, *J* = 6.1 Hz, 1H), 3.92 (s, 3H), 1.43 (d, *J* = 6.1 Hz, 6H); IR (cm^−1^) 1696, 1593, 1506, 1284, 1151. Spectral data are in accordance with ref. 69.

### Production of ketones 7b–d by Darzens reaction^70^

#### General method

To a solution of the corresponding aldehyde (9b–d, 6 mmol) in toluene (10 mL) at room temperature was added (±)-2-chloropropionic acid methyl ester (7.2 mmol, 1.2 equiv.) dropwise. The solution was cooled to 0–5 °C and a freshly prepared solution of NaOMe in MeOH (1.15 equiv., 150 mg Na in 1.7 mL MeOH, 25 w/w%) was added at a rate that the temperature of the mildly exothermic remained below 10 °C. The suspension was stirred for an additional 20 min at 0–5 °C, and then warmed to room temperature and stirred until completion of the reaction (24 h). After addition of 1 M aqueous NaOH (5 mL) to the resulted mixture, stirring was performed at 50 °C for 4 h, followed by another period at room temperature overnight. Then, water (20 mL) and toluene (10 mL) were added, and the aqueous phase was extracted with toluene (2 × 20 mL). 5 mL 5 M aqueous HCl was added (pH 2–3) and the biphasic mixture was refluxed at 80–85 °C for 2 hours. The organic phase was washed with water (10 mL), dried over sodium sulphate and concentrated under vacuum. The residue was purified by flash chromatography over silica gel (eluent: hexane/ethyl acetate 7 : 3) to give the desired ketone (7b–d).

#### 1-(3,4-Dimethoxyphenyl)propan-2-one (7b)

Yellow oil, TLC: *R*_f_ = 0.5. Yield: 59%. ^1^H-NMR (500 MHz, CDCl_3_) 6.82 (d, *J* = 8.2 Hz, 1H), 6.74 (dd, *J* = 8.2, 2.0 Hz, 1H), 6.69 (d, *J* = 2.0 Hz, 1H), 3.85 (s, 6H), 3.62 (s, 2H), 2.14 (s, 3H); ^13^C-NMR (126 MHz, CDCl_3_) 206.77, 149.12, 148.17, 126.74, 121.58, 112.39, 111.43, 55.91, 55.88, 50.61, 29.08; IR (cm^−1^) 1707, 1591, 1513, 1464, 1453, 1259, 1235, 1154, 1138, 1024; HRMS (ESI/Q-TOF) *m*/*z*: [M + H]^+^ calculated for C_11_H_15_O_3_ 195.1021, found 195.1031. Spectral data are in accordance with ref. 30.

#### 1-(4-Ethoxy-3-methoxyphenyl)propan-2-one (7c)

Yellow oil, TLC: *R*_f_ = 0.4. Yield: 79%. ^1^H-NMR (500 MHz, CDCl_3_) 6.82 (d, *J* = 8.1 Hz, 1H), 6.71 (dd, *J* = 8.1, 2.1 Hz, 1H), 6.69 (d, *J* = 2.0 Hz, 1H), 4.07 (q, *J* = 7.0 Hz, 2H), 3.84 (s, 3H), 3.61 (s, 2H), 2.13 (s, 2H), 1.44 (t, *J* = 7.0 Hz, 3H); ^13^C-NMR (126 MHz, CDCl_3_) 206.89, 149.42, 147.47, 126.70, 121.57, 112.89, 112.65, 64.34, 55.94, 50.65, 29.07, 14.82; IR (cm^−1^) 1708, 1590, 1511, 1420, 1258, 1228, 1156, 1139, 1032; HRMS (ESI/Q-TOF) *m*/*z*: [M + H]^+^ calculated for C_12_H_17_O_3_ 209.1178, found 209.1191.

#### 1-(4-Isopropoxy-3-methoxyphenyl)propan-2-one (7d)

Yellow oil, TLC: *R*_f_ = 0.5. Yield: 47%. ^1^H-NMR (300 MHz, CDCl_3_) 6.86 (d, *J* = 8.4 Hz, 1H), 6.73 (d, *J* = 6.2 Hz, 2H), 4.51 (m, *J* = 6.1 Hz, 1H), 3.85 (s, 3H), 3.63 (s, 2H), 2.16 (s, 3H), 2.14 (d, *J* = 7.4 Hz, 0H), 1.37 (dd, *J* = 6.1, 1.7 Hz, 6H); ^13^C-NMR (75 MHz, CDCl_3_) 206.87, 150.56, 146.49, 127.17, 121.59, 116.07, 113.15, 71.53, 55.99, 50.69, 29.11, 22.14; IR (cm^−1^) 1707, 1590, 1511, 1452, 1156, 1139, 1032; HRMS (ESI/Q-TOF) *m*/*z*: [M + H]^+^ calculated for C_13_H_19_O_3_ 223.1334, found 223.1334.

### Synthesis of racemic amines 8a–d by reductive amination^71,72^

#### General method

To the solution of ketone 7a–d (1 mmol) and ammonium formate (10 mmol, 10 equiv.) in methanol (5 mL) containing water (0.5 mL) was added 10% Pd/C (0.04 equiv. of Pd). The mixture was stirred overnight at room temperature. After the reaction completed, the mixture was filtered through Celite, washed with methanol and the solution was concentrated under vacuum. To the residue aq. HCl (37% w/w, 2 mL) and water (10 mL) were added, and the aqueous phase was extracted with diethyl ether (2 × 10 mL). The aqueous phase was then adjusted to pH 10 with aq. NH_3_ (35% w/w) solution and extracted with dichloromethane (4 × 15 mL). The unified organic phases were washed with brine (15 mL) and dried over anhydrous sodium sulphate, then concentrated under vacuum. The resulted product was used in further steps as such.

#### 1-Phenylpropan-2-amine (8a)

Yellow oil. Yield: 70%. ^1^H-NMR (300 MHz, DMSO-*d*_6_) 7.28 (t, *J* = 7.4 Hz, 2H), 7.17 (d, *J* = 7.5 Hz, 3H), 2.99 (m, 1H), 2.52 (d, *J* = 7.2 Hz, 2H), 1.58 (s, 2H), 0.95 (d, *J* = 6.2 Hz, 3H); ^13^C-NMR (75 MHz, DMSO-*d*_6_) 140.59, 129.61, 128.60, 126.27, 48.78, 46.78, 23.79; IR (cm^−1^) 3363, 3026, 1583, 1495, 1452, 1370, 1088; HRMS (ESI/Q-TOF) *m*/*z*: [M + H]^+^ calculated for C_9_H_14_N 136.1126, found 136.1123. Spectral data are in accordance with ref. 71.

#### 1-(3,4-Dimethoxyphenyl)propan-2-amine (8b)

Yellow oil. Yield: 61%. ^1^H-NMR (500 MHz, CDCl_3_) 6.81 (d, *J* = 7.9 Hz, 1H), 6.76–6.66 (m, 2H), 3.87 (s, 3H), 3.86 (s, 3H), 3.20–3.09 (m, 1H), 2.67 (dd, *J* = 13.4, 5.2 Hz, 1H), 2.51–2.33 (m, 2H), 1.63 (s, 1H), 1.12 (d, *J* = 6.3 Hz, 3H); ^13^C-NMR (126 MHz, CDCl_3_) 148.83, 147.47, 132.30, 121.14, 112.41, 111.24, 55.91, 55.83, 48.55, 46.14, 23.51; IR (cm^−1^) 3344, 1589, 1512, 1463, 1417, 1259, 1234, 1138, 1025; HRMS (ESI/Q-TOF) *m*/*z*: [M + H]^+^ calculated for C_11_H_18_NO_2_: 196.1339, found 196.1336. Spectral data are in accordance with ref. 30.

#### 1-(4-Ethoxy-3-methoxyphenyl)propan-2-amine (8c)

Yellow oil. Yield: 55%. ^1^H-NMR (500 MHz, CDCl_3_) 6.74 (d, *J* = 8.0 Hz, 1H), 6.64 (d, *J* = 7.3 Hz, 2H), 4.01 (q, *J* = 7.0 Hz, 2H), 3.79 (s, 3H), 3.12–3.02 (m, 1H), 2.59 (dd, *J* = 13.4, 5.2 Hz, 1H), 2.37 (dd, *J* = 13.4, 8.3 Hz, 1H), 1.38 (t, *J* = 7.0 Hz, 3H), 1.05 (d, *J* = 6.3 Hz, 3H); ^13^C-NMR (126 MHz, CDCl_3_) 149.15, 146.74, 132.32, 121.14, 112.82, 112.70, 64.35, 55.90, 48.53, 46.19, 23.54, 14.87; IR (cm^−1^) 3359, 1588, 1513, 1259, 1234, 1139, 1091, 1031; HRMS (ESI/Q-TOF) *m*/*z*: [M + H]^+^ calculated for C_12_H_20_NO_2_ 210.1494, found 210.1494.

#### 1-(4-Isopropoxy-3-methoxyphenyl)propan-2-amine (8d)

Yellow oil. Yield: 64%; ^1^H-NMR (500 MHz, CDCl_3_) 6.87 (d, *J* = 8.1 Hz, 1H), 6.81 (d, *J* = 2.0 Hz, 1H), 6.71 (dd, *J* = 8.1, 2.0 Hz, 1H), 4.47 (p, *J* = 6.1 Hz, 1H), 3.81 (s, 3H), 3.13–3.02 (m, 1H), 2.60 (dd, *J* = 13.3, 6.2 Hz, 1H), 2.53 (dd, *J* = 13.3, 7.5 Hz, 1H), 1.28 (d, *J* = 6.1 Hz, 6H), 1.08 (d, *J* = 6.3 Hz, 3H); ^13^C-NMR (126 MHz, CDCl_3_) 152.08, 146.87, 134.57, 122.54, 118.47, 114.76, 73.22, 56.41, 46.39, 22.68, 22.43, 15.04. IR (cm^−1^) 3352, 1588, 1511, 1452, 1259, 1234, 1138, 1091, 1026; HRMS (ESI/Q-TOF) *m*/*z*: [M + H]^+^ calculated for C_13_H_22_NO_2_ 224.1651, found 224.1640.

### Biotransformations

Statistical analysis on the conversion of the unsubstituted ketone 7a in transamination reaction could be performed from 5 points: 83.0% (Table 1), 85.5% (Fig. 3), 88.2% (Fig. 4), 88.9% (Table 2), 87.4% (Fig. 5). Based on these data, standard deviation of the population could be calculated as 2.1% for *μ* = 86.6% as the mean value. Since the SD of conversion was quite moderate in biotransformation of 7a, the further investigations were performed as single sets of experiments.

#### Kinetic resolution of racemic amines 8a–d

##### General method

To a 4 mL screw-capped vial containing KPi buffer (890 μL, 100 mM, pH 7.5) were added immobilized whole-cell TA (20 mg), PLP solution (10 μL, 100 mM in KPi buffer), DMSO solution of the racemic amine (50 μL, 200 mM of 8a–d in DMSO), and sodium pyruvate solution (50 μL, 100 mM in KPi buffer) and the resulted mixture was shaken at 450 rpm at 30 °C for 24 h. Then, the reaction was terminated by the addition of 1 M aqueous NaOH solution (100 μL) and EtOAc (500 μL). The aqueous phase was separated, and the organic phases was dried over anhydrous sodium sulphate. The conversion from the racemic amine (8a–d) to ketone (7a–d) and the enantiomeric excess of the unreacted amine [(*S*)-8a–d] were determined by chiral GC [50 μL of extracted reaction sample was derivatised to the corresponding acetamides (by addition of 10 μL acetic anhydride and shaking at 60 °C by 750 rpm for 1 h)]. The results are summarized in Fig. 6.

#### Asymmetric synthesis of (*R*)-8a–d

##### General method

To a 4 mL screw-capped vial containing KPi buffer (930 μL, 100 mM, pH 7.5) were added immobilized whole-cell TA (20 mg), PLP solution (10 μL, 100 mM in KPi buffer), DMSO solution of the ketone (50 μL, 200 mM of 7a–d in DMSO), and *sec*-butylamine 12 (10.1 μL, equal to 100 mM in the final volume), and the pH 8 was set with 1 M aqueous HCl solution. Then, the resulted mixture was shaken at 450 rpm and 30 °C for 24 h, and the reactions were terminated by the addition of 1 M aqueous NaOH solution (100 μL) and EtOAc (500 μL). The aqueous phase was separated, and the organic phases was dried over anhydrous sodium sulphate. The conversion from the ketone (7a–d) and the enantiomeric excess of the formed amine [(*R*)-8a–d] were determined by chiral GC [50 μL of extracted reaction sample was derivatised to the corresponding acetamides (by addition of 10 μL acetic anhydride and shaking at 60 °C by 750 rpm for 1 h)]. The results are summarized in Table 2.

## Conclusions

This study with three (*R*)-selective transaminases of *Arthrobacter* sp. and *Aspergillus terreus* applied as immobilized whole-cell biocatalysts of the overexpressing *E. coli* cells showed that enantiopure drug-like (*R*)-1-(3′,4′-disubstituted phenyl)propan-2-amines (*R*)-8a–d could be obtained from the corresponding 1-arylpropan-2-ones (7a–d) by asymmetric transamination, while kinetic resolution with these (*R*)-selective TAs could provide the (*S*)-amines (*S*)-8a–d from the corresponding racemic amines 8a–d.

The 3′,4′-disubstituted 1-phenylpropan-2-ones (7b–d) have been synthesised conveniently from vanillin (10) in two steps. The high-yield *O*-alkylations of 10 followed by Darzens reaction gave the desired ketones 7b–d in good yields. The racemic amines (8b–d) were obtained by Pd-catalysed reductive amination of 7b–d.

The reaction optimisation on model compound 7a probing seven different amine donors, and the effect of the substrate and co-solvent DMSO concentration revealed *sec*-butylamine as the most suitable amine donor. While high conversions with Ar*R*-TA and At*R*-TA with excellent selectivity (ee > 99%) could be achieved only up to 10 mM substrate concentration, the highly engineered Ar*R*_m_-TA provided sufficiently high conversion even at 100 mM, albeit with insufficient selectivity (ee ∼ 80%).

With Ar*R*-TA as the most versatile biocatalyst in this study, the (*R*)-1-phenylpropan-2-amine (8a) was obtained with 92% conversion and >99% ee employing *sec*-butylamine as amine donor under the optimised reaction conditions in 24 h. Ar*R*-TA catalysed the transamination of all the 3,4-disubstituted ketones 7b–d as well with equally high conversion (88–89%) and excellent selectivity (ee > 99%). In addition, the kinetic resolution of 8a,c,d with At*R*-TA could provide the (*S*)-amines (*S*)-8a–d with perfect conversion (>49% in KR) and good selectivity (ee > 95%), only the KR of 8b proceeded with lower conversion (c = 48.2%) and moderate selectivity (ee = 92%).

## Conflicts of interest

There are no conflicts to declare.

## Supplementary Material

RA-010-D0RA08134E-s001
