# Supplementary material for: Transaminase-mediated synthesis of enantiopure drug-like 1-(3′,4′-disubstituted phenyl)propan-2-amines
Source: RSC Adv. 2020 Nov 10;10(67):40894–903. doi: 10.1039/d0ra08134e (PMC9057730; doi:10.1039/d0ra08134e)
Supplement: RA-010-D0RA08134E-s001 [file RA-010-D0RA08134E-s001.pdf]

Electronic Supplementary Material (ESI) for RSC Advances.
This journal is © The Royal Society of Chemistry 2020

Electronic Supplementary Information

Transaminase-mediated synthesis of enantiopure drug-like 1-(3',4'-disubstituted phenyl)propan-2-amines

Ágnes Lakó, Zsófia Molnár, Ricardo Mendonça and László Poppe

Table of Contents

1. GC monitoring of the enzymatic reactions **2**

1.1 Determination of the conversion and enantiomeric excess by GC [Table S1] **2**

1.2 GC chromatograms of the reference compounds and of the products of TA-catalyzed stereoselective biotransformations [Fig. S1-S16] **2**

2. NMR spectra of the synthetic aldehydes (10b-d), ketones (7b-d), and amines (8a-d) [Fig. S17-S33] **7**

1. GC monitoring of the enzymatic reactions

1.1. Determination of the conversion and enantiomeric excess by GC

Gas chromatographic (GC) analyses were performed with an Agilent 4890 gas chromatograph equipped with FID detector using H_2 carrier gas (injector: 250 °C, detector: 250 °C, head pressure: 12 psi, split ratio: 50:1) and Hydrodex β-6TBDM column [25 m×0.25 mm×0.25 µm film with heptakis-(2,3-di-O-methyl-6-O-t-butyldimethylsilyl)-β-cyclodextrine; Macherey & Nagel].
Conversion (*c*) and enantiomeric excess values (*ee*) were determined by GC measurements with base-line separations of the peaks for the enantiomers of racemic amines **8a-d** as acetamides **8*a-d** (for details see the **Calculations** section of the main text).

Table S1. GC methods, retention times, and response factors, used for the conversion value determinations by quantitative GC analysis

Substrate	Temperature program	Retention times (min)			Response factors
		Ketone **7**	(*S*)-amine (*S*)-**8***	(*R*)-amine (*R*)-**8***	**8*** vs. **7**
**7a**	110 °C hold 20 min, 5 °C min^{-1} to 190 °C	8.14	32.05	32.32	1.11
**7b**	160°C, 0.8 °C min^{-1} to 190°C, 1 min hold	8.83	31.22	31.64	0.86
**7c**		10.07	34.00	34.41	0.90
**7d**		10.24	34.24	34.75	1.00

*Enantiomers of racemic amines **8a-d** were separated as their acetamides **8*a-d**

1.2 GC chromatograms of the reference compounds and of the products of TA-catalyzed stereoselective biotransformations

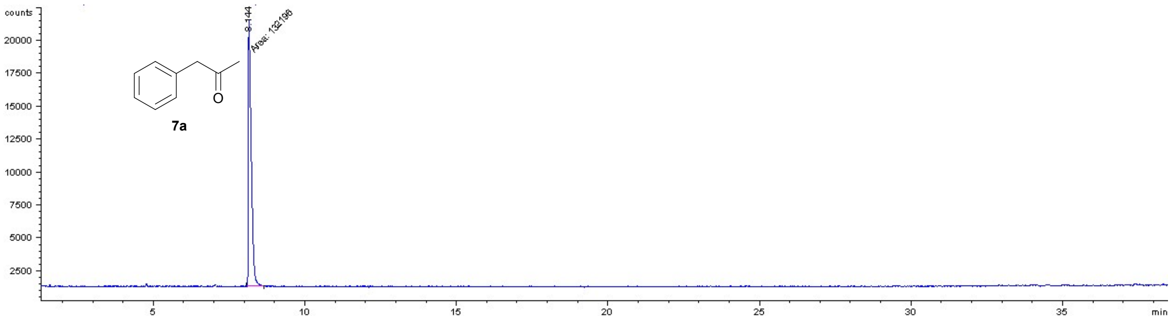

Figure S1. GC chromatogram of 1-phenylpropan-2-one (**7a**).

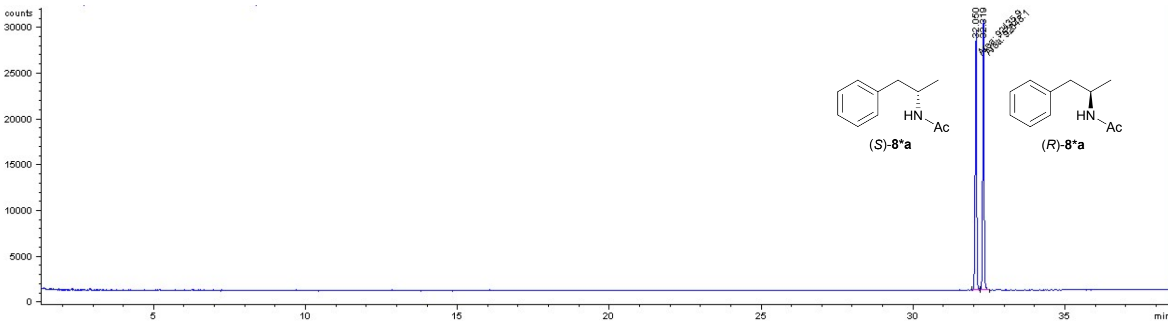

Figure S2. GC chromatogram of acetamide (**8*a**) from racemic 1-phenylpropan-2-amine (**8a**) after derivatization with Ac_2O.

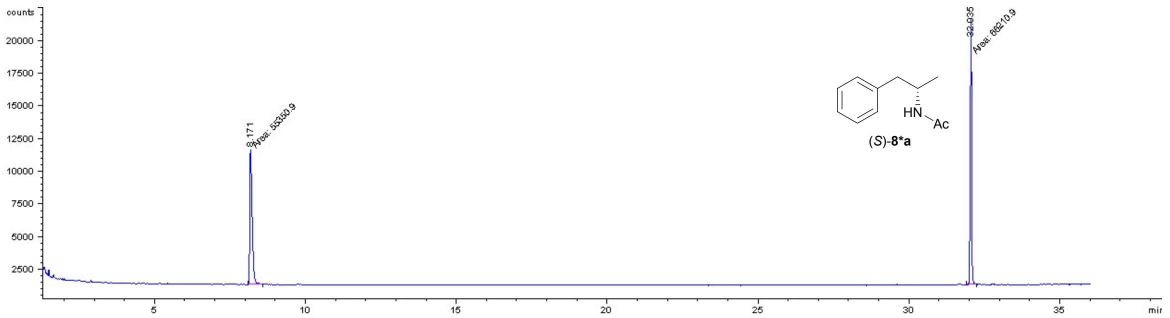

Figure S3. GC chromatogram of the product of kinetic resolution from racemic of 1-phenylpropan-2-amine (**8a**) after derivatization with Ac_2O.

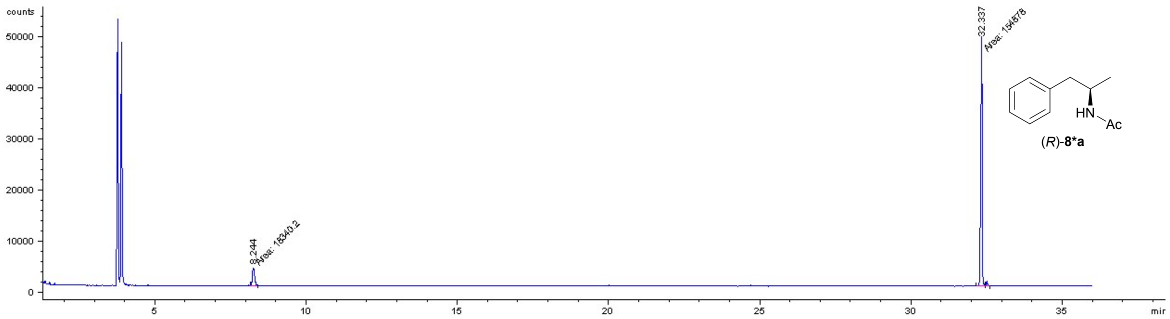

Figure S4. GC chromatogram of the product of asymmetric amination from 1-phenylpropan-2-one (**7a**) after derivatization with Ac_2O.

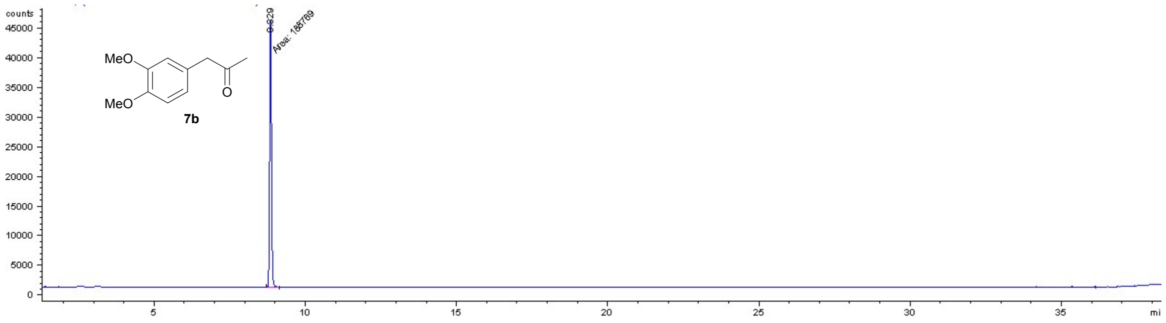

Figure S5. GC chromatogram of 1-(3,4-dimethoxyphenyl)propan-2-one (**7b**).

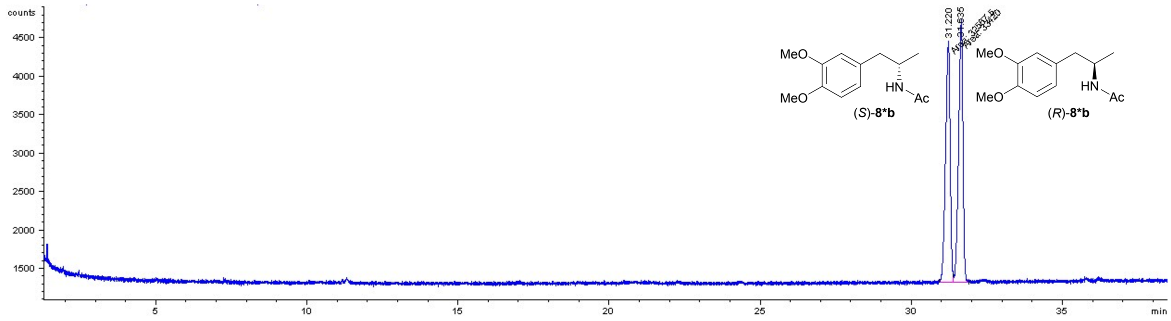

Figure S6. GC chromatogram of acetamide (**8*b**) from racemic 1-(3,4-dimethoxyphenyl)propan-2-amine (**8b**) after derivatization with Ac_2O.

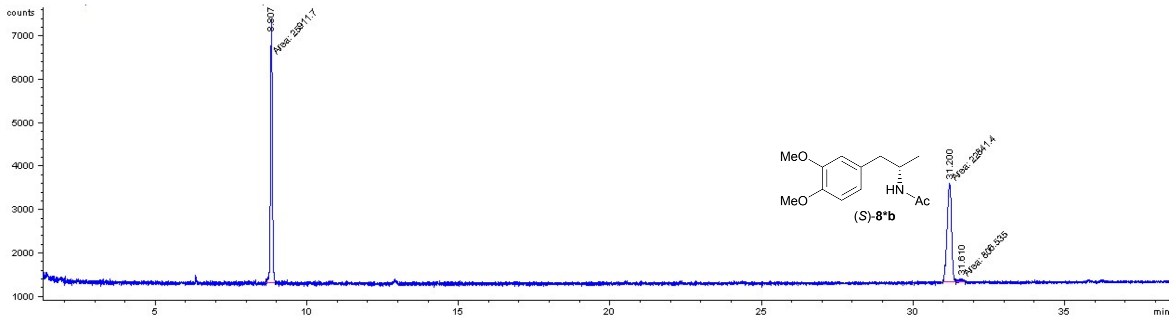

Figure S7. GC chromatogram of the product of kinetic resolution from racemic 1-(3,4-dimethoxyphenyl)propan-2-amine (**8b**) after derivatization with Ac_2O.

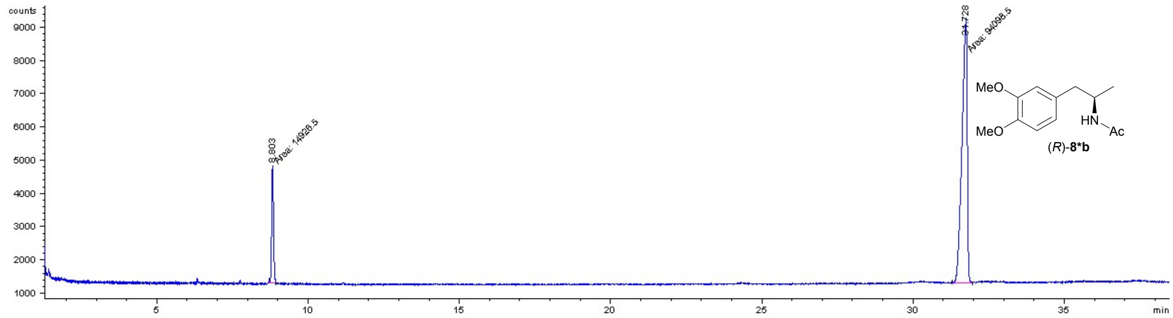

Figure S8. GC chromatogram of the product of asymmetric amination from 1-(3,4-dimethoxyphenyl)propan-2-one (**7b**) after derivatization with Ac_2O.

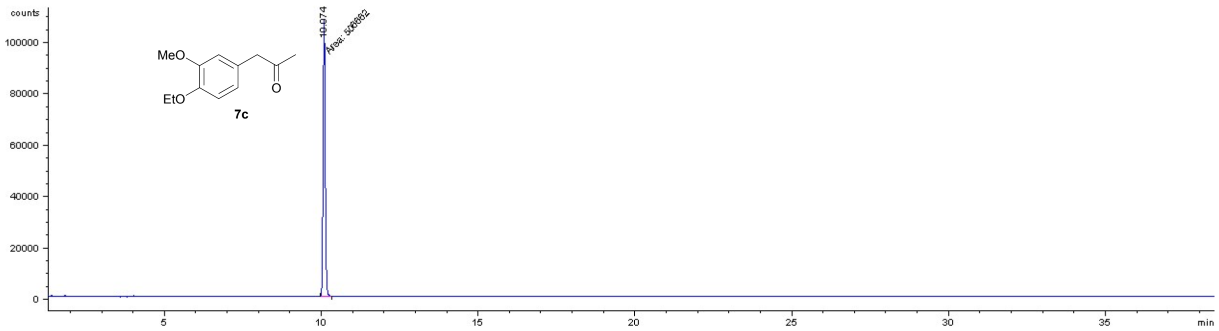

Figure S9. GC chromatogram of 1-(4-ethoxy-3-methoxyphenyl)propan-2-one (**7c**).

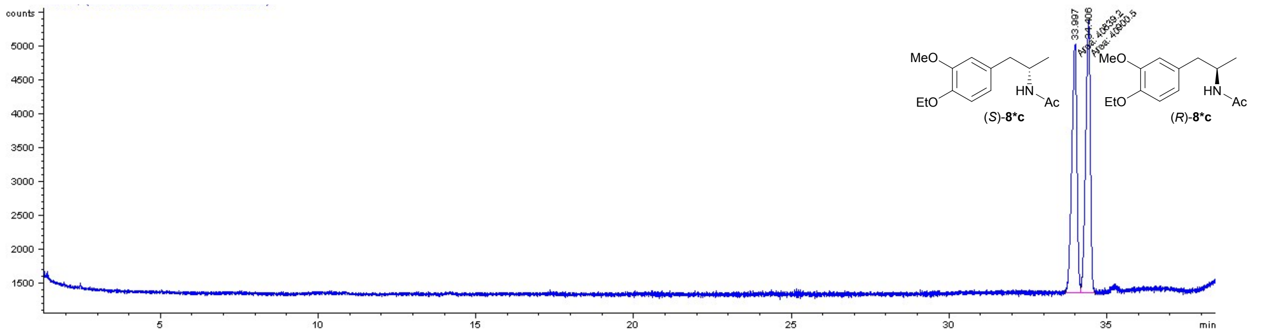

Figure S10. GC chromatogram of acetamide (**8*c**) from racemic 1-(4-ethoxy-3-methoxyphenyl)propan-2-amine (**8c**) after derivatization with Ac_2O.

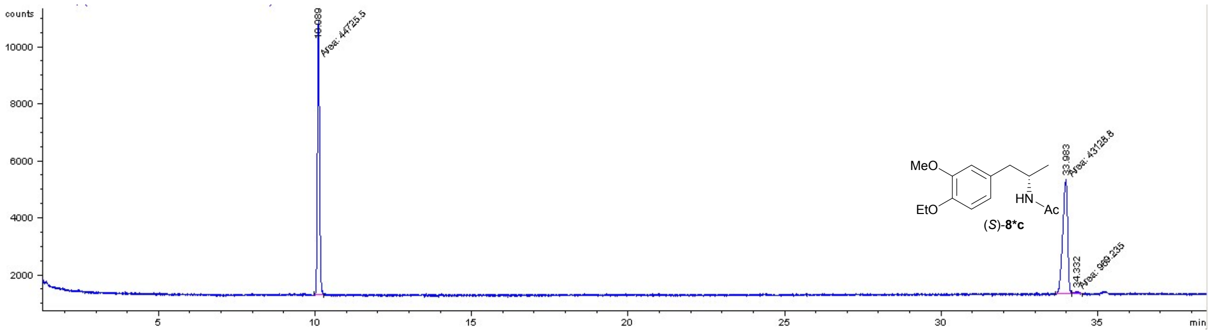

Figure S11. GC chromatogram of the product of kinetic resolution from racemic 1-(4-ethoxy-3-methoxyphenyl)propan-2-amine (**8c**) after derivatization with Ac_2O.

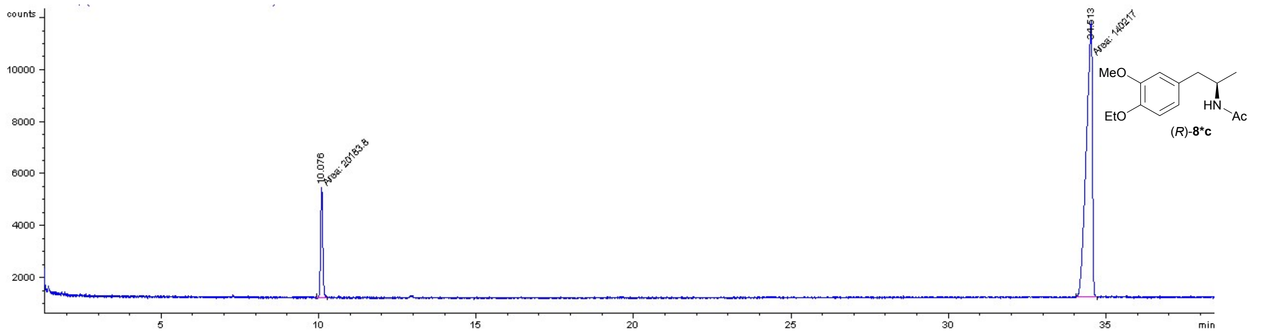

Figure S12. GC chromatogram of the product of asymmetric amination from 1-(4-ethoxy-3-methoxyphenyl)propan-2-one (**7c**) after derivatization with Ac_2O.

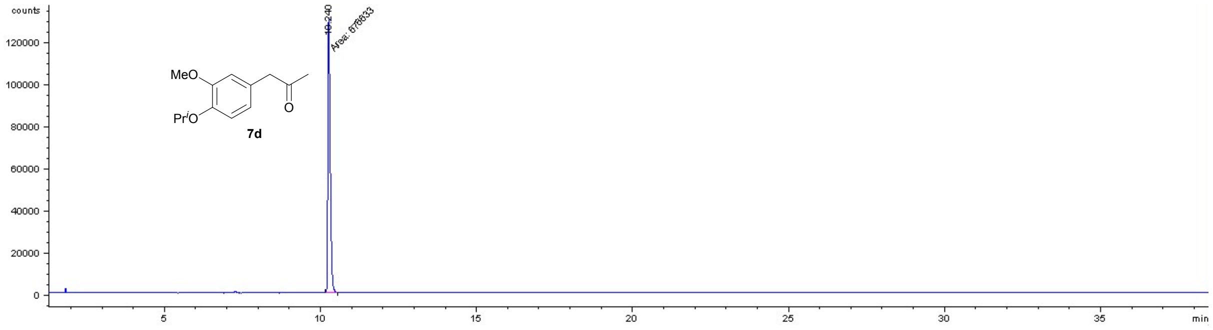

Figure S13. GC chromatogram of 1-(4-isopropoxy-3-methoxyphenyl)propan-2-one (**7d**).

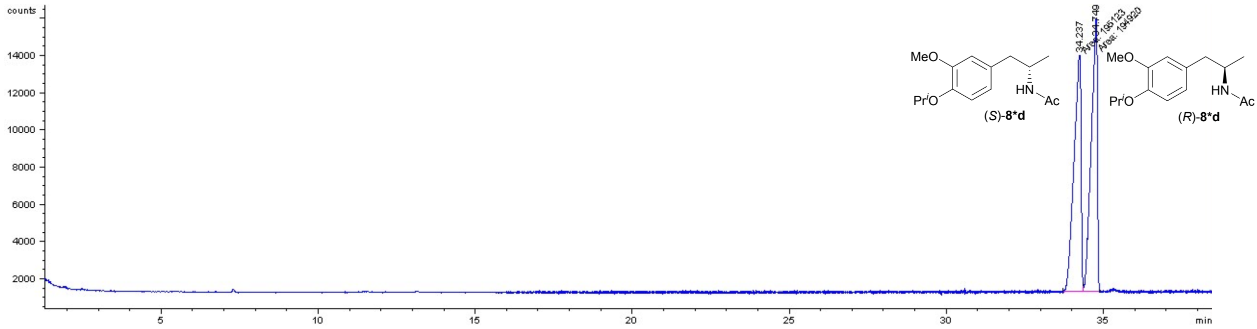

Figure S14. GC chromatogram of acetamide (**8*c**) from racemic 1-(4-isopropoxy-3-methoxyphenyl)propan-2-amine (**8d**) after derivatization with Ac_2O.

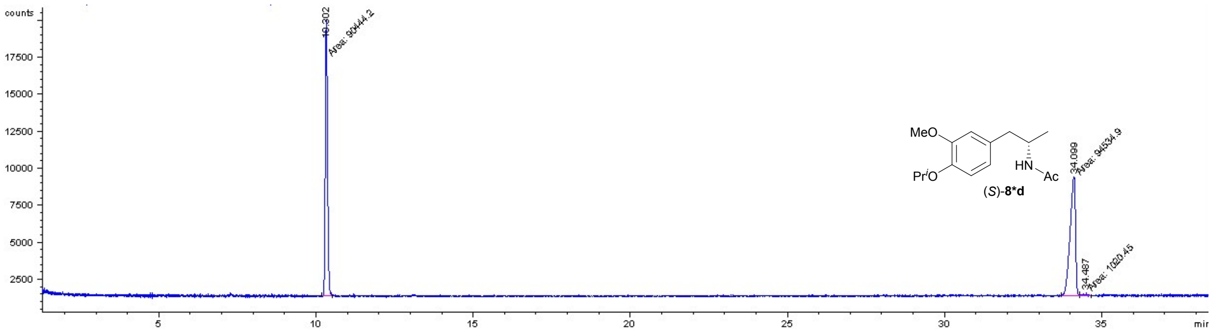

Figure S15. GC chromatogram of the product of kinetic resolution from racemic 1-(4-isopropoxy-3-methoxyphenyl)propan-2-amine (**8d**) after derivatization with Ac_2O.

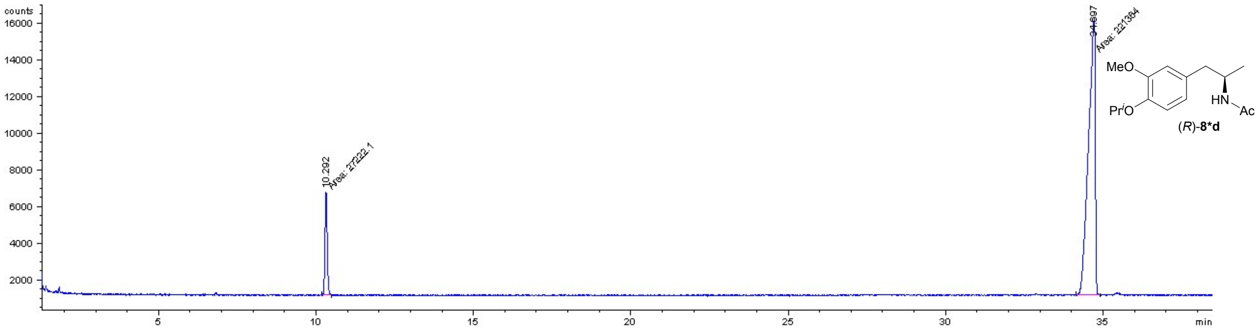

Figure S16. GC chromatogram of the product of asymmetric amination from 1-(4-isopropoxy-3-methoxyphenyl)propan-2-one (**7d**) after derivatization with Ac_2O.

2. NMR spectra of the synthetic aldehydes (10b-d), ketones (7b-d), and amines (8a-d)

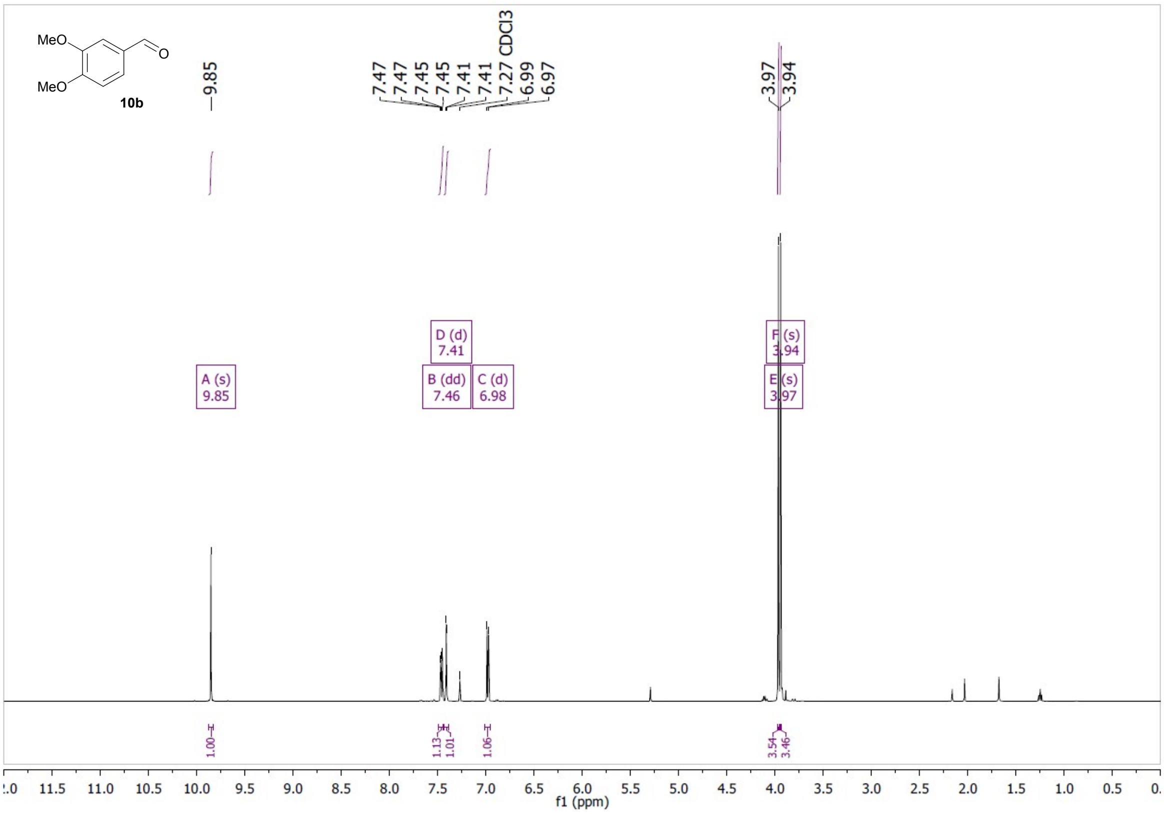

Figure S17. ^{1}H-NMR spectrum of 3,4-dimethoxybenzaldehyde (**10b**)

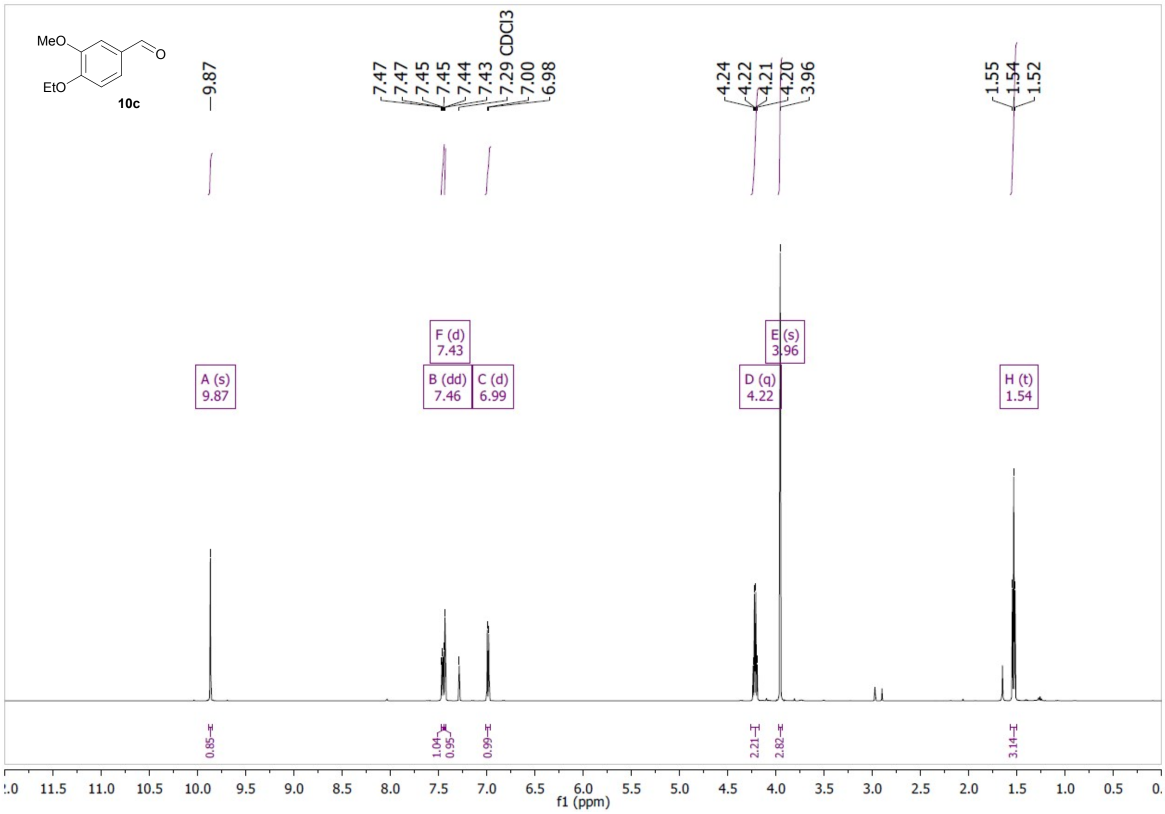

Figure S18. ^{1}H-NMR spectrum of 4-ethoxy-3-methoxybenzaldehyde (**10c**)

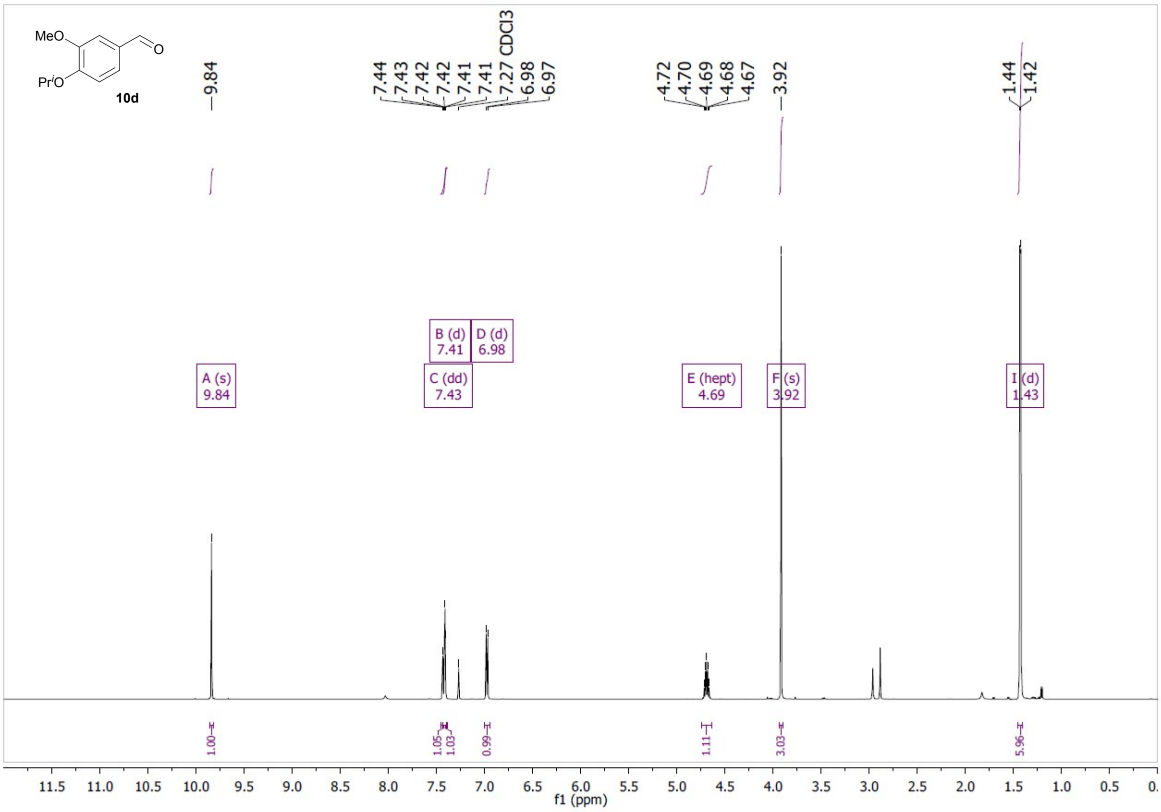

Figure S19. ^{1}H-NMR spectrum of 4-isopropoxy-3-methoxybenzaldehyde (**10d**)

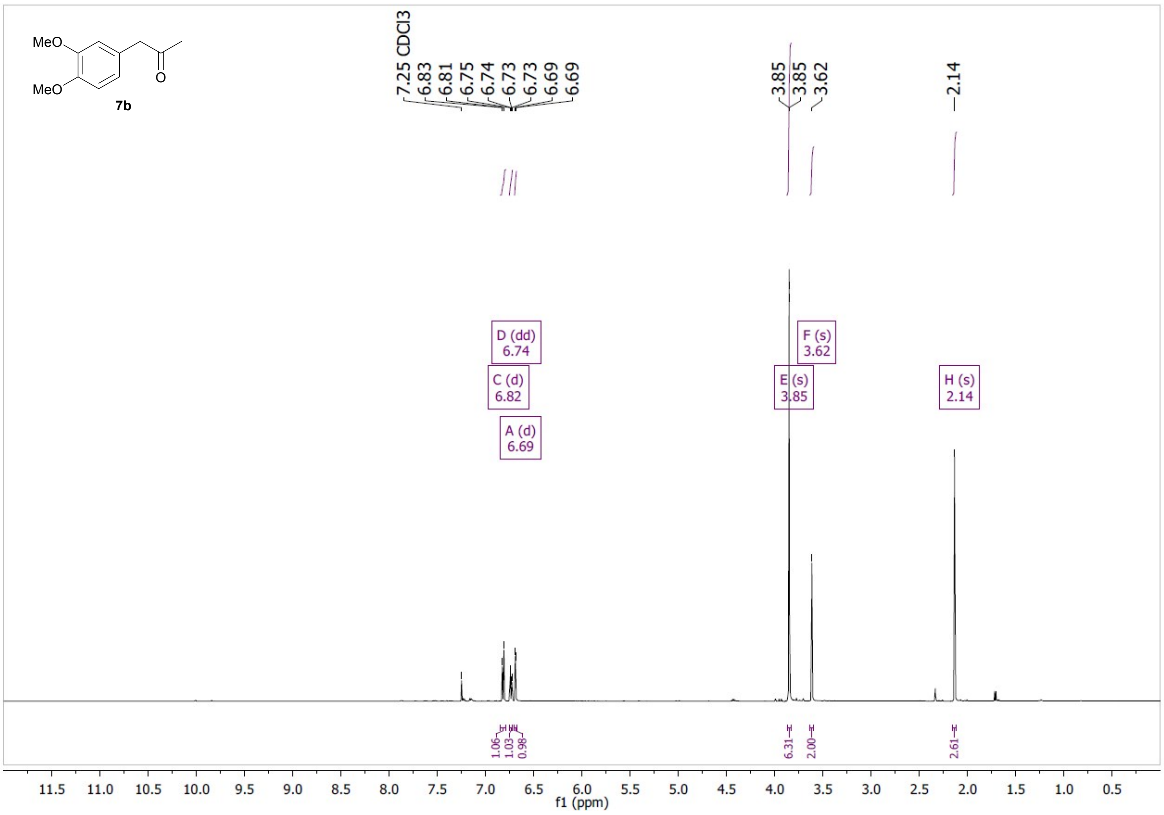

Figure S20. ^{1}H-NMR spectrum of 1-(3,4-dimethoxyphenyl)propan-2-one (**7b**)

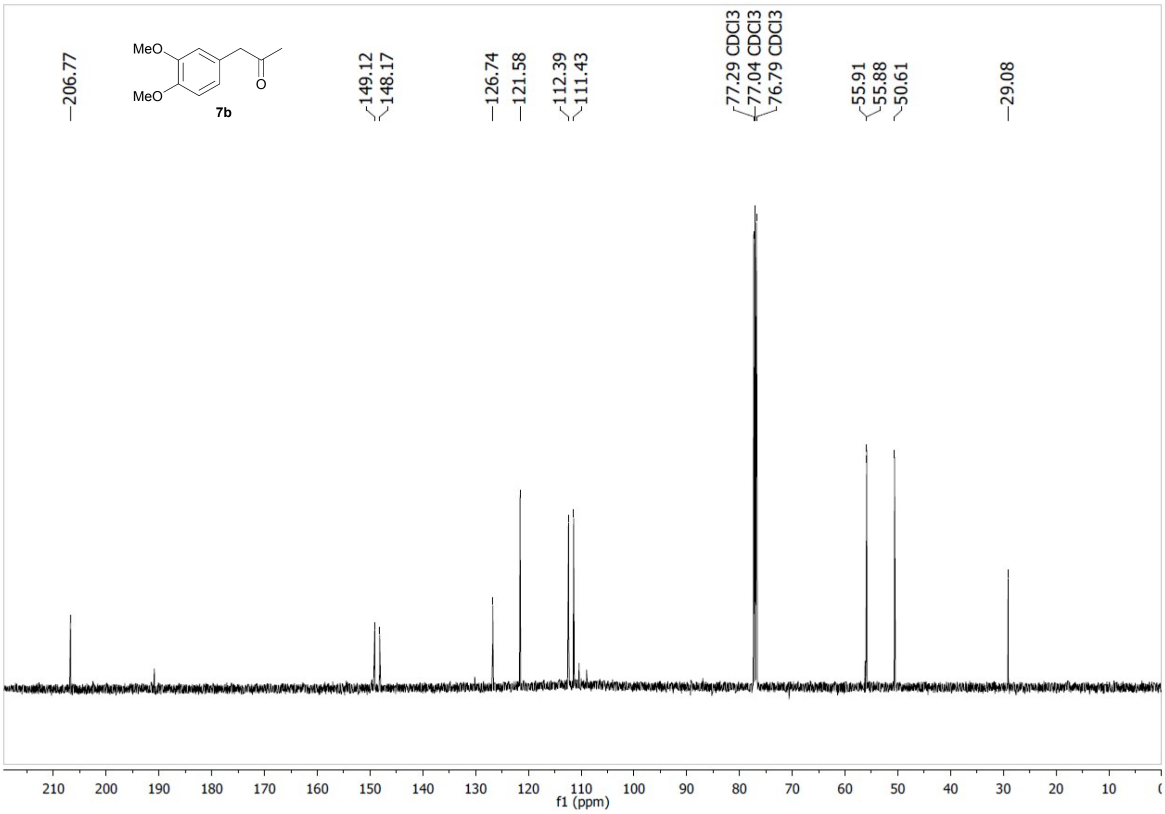

Figure S21. ^{13}C-NMR spectrum of 1-(3,4-dimethoxyphenyl)propan-2-one (**7b**)

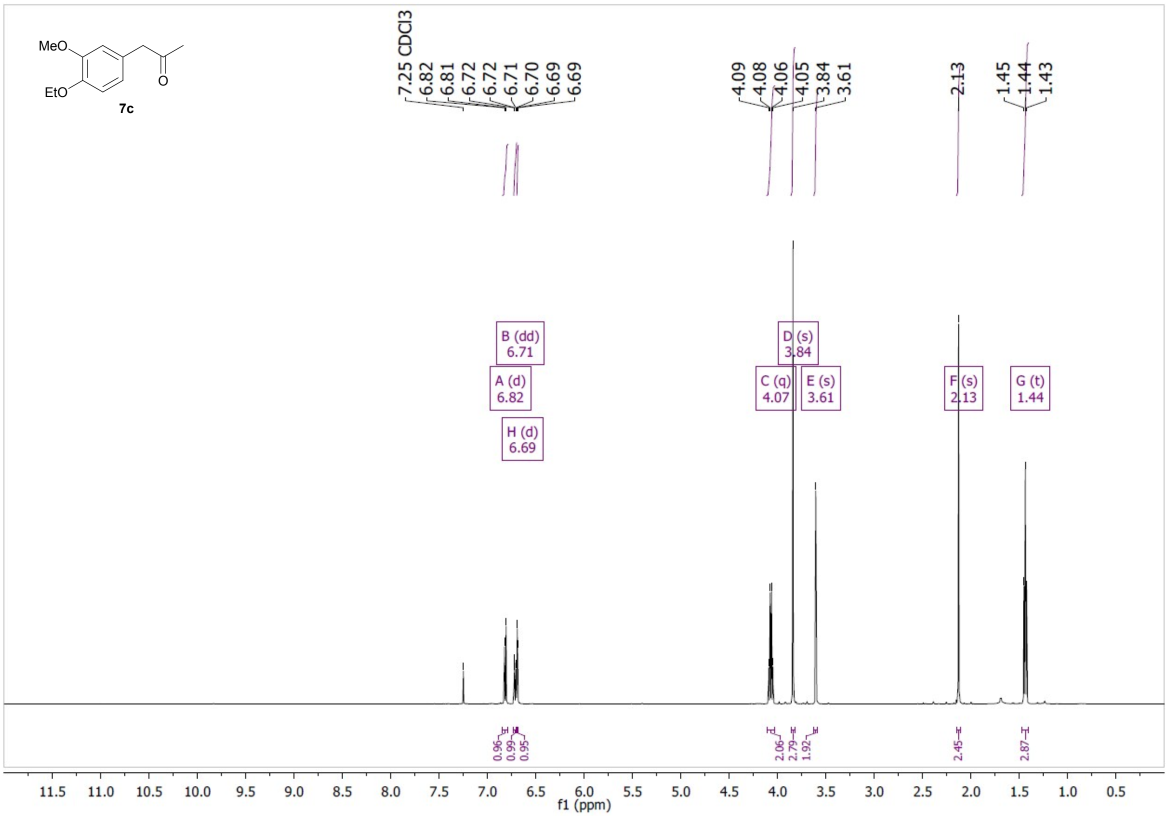

Figure S22. ^{1}H-NMR spectrum of 1-(4-ethoxy-3-methoxyphenyl)propan-2-one (**7c**)

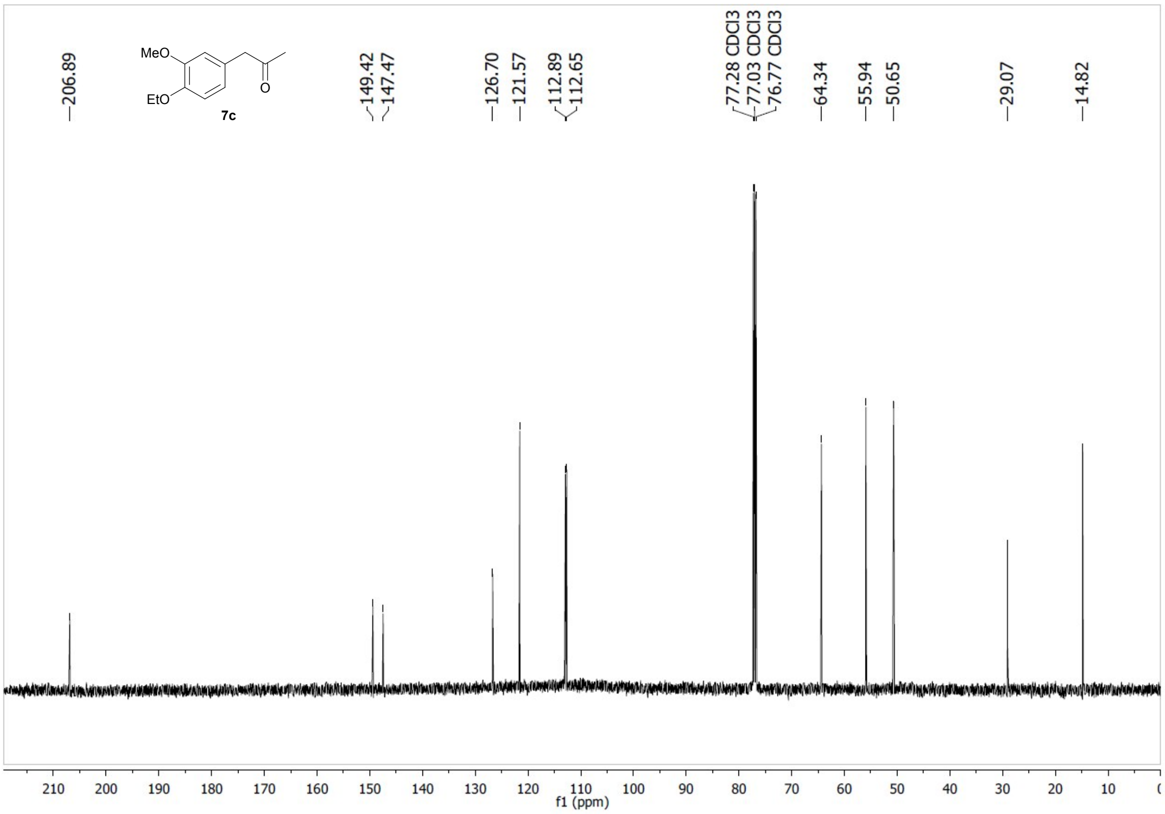

Figure S23. ^{13}C-NMR spectrum of 1-(4-ethoxy-3-methoxyphenyl)propan-2-one (**7c**)

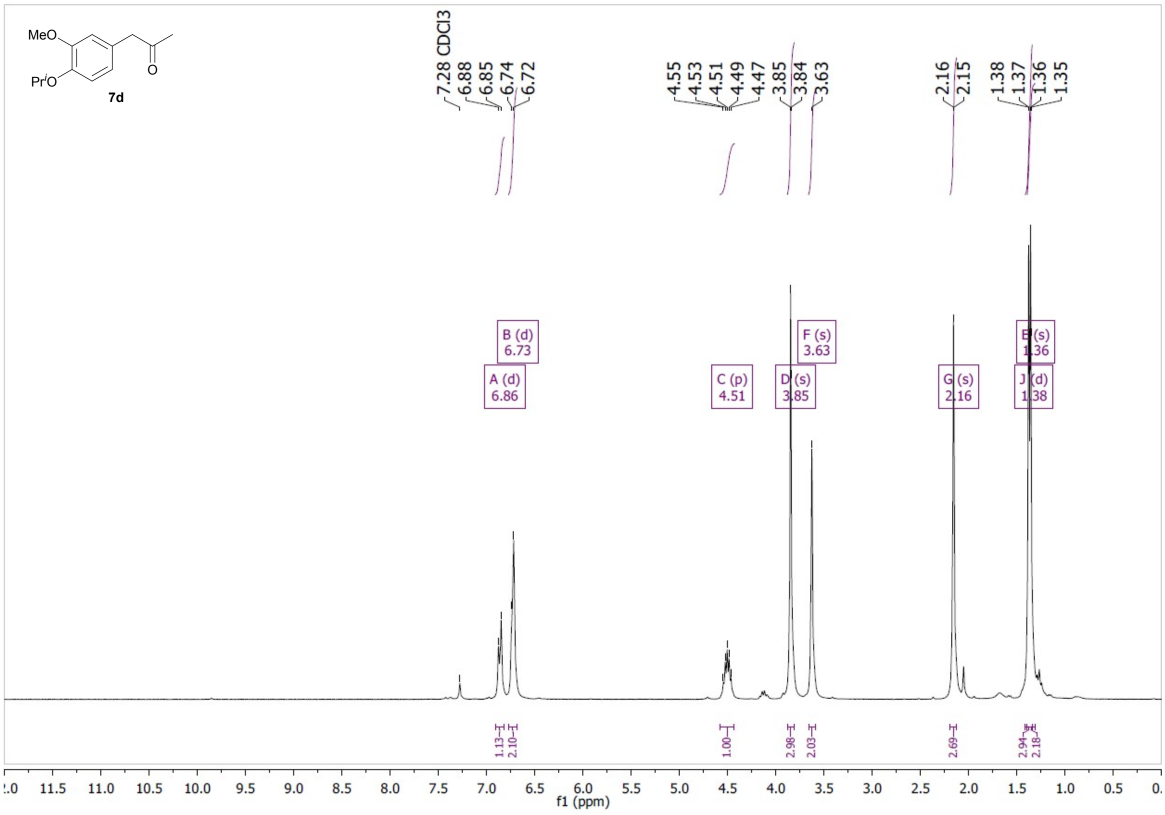

Figure S24. ^{1}H-NMR spectrum of 1-(4-isopropoxy-3-methoxyphenyl)propan-2-one (**7d**)

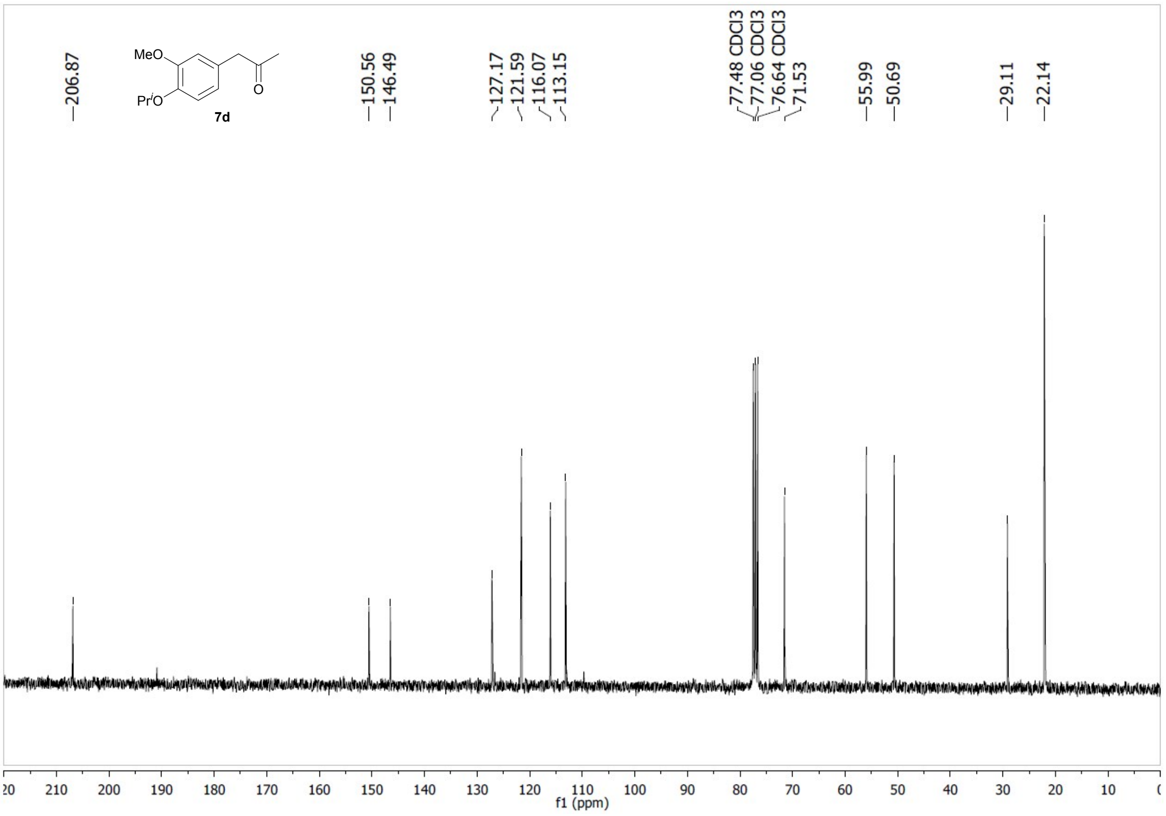

Figure S25. ^{13}C-NMR spectrum of 1-(4-isopropoxy-3-methoxyphenyl)propan-2-one (**7d**)

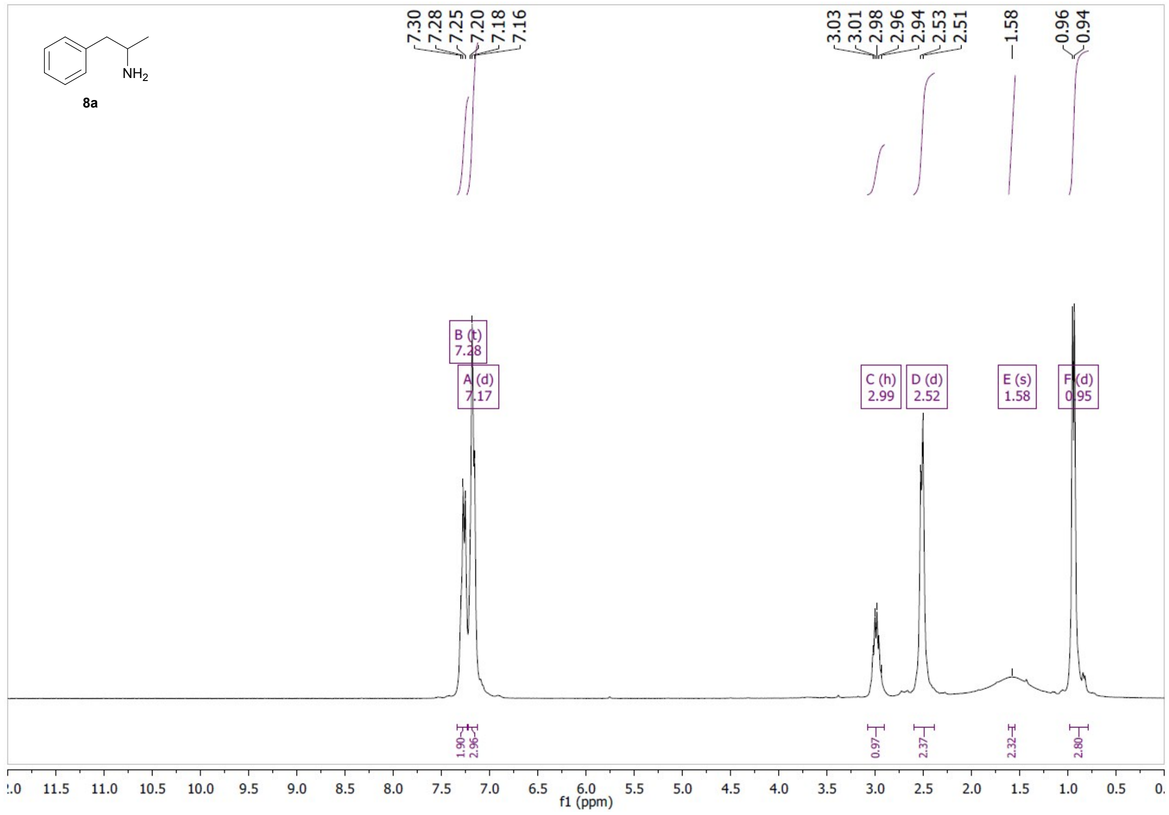

Figure S26. ^{1}H-NMR spectrum of 1-phenylpropan-2-amine (**8a**)

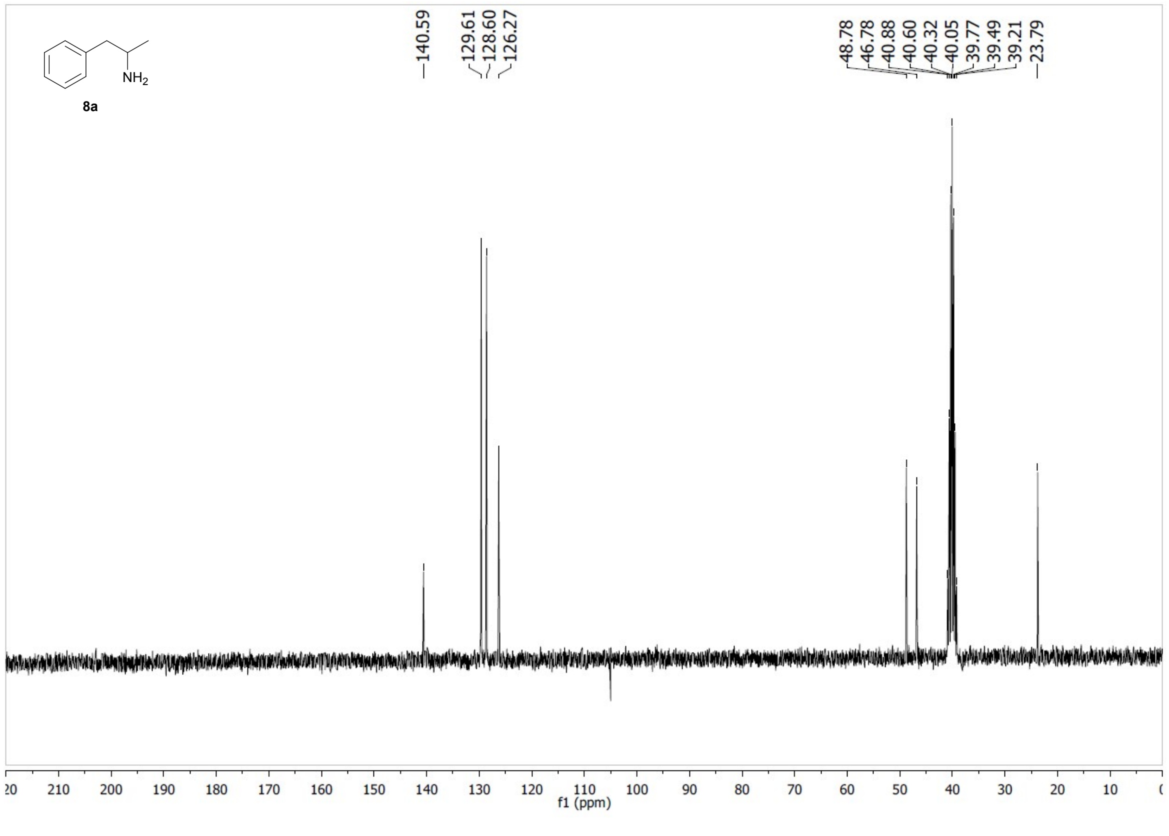

Figure S27. ^{13}C-NMR spectrum of 1-phenylpropan-2-amine (**8a**)

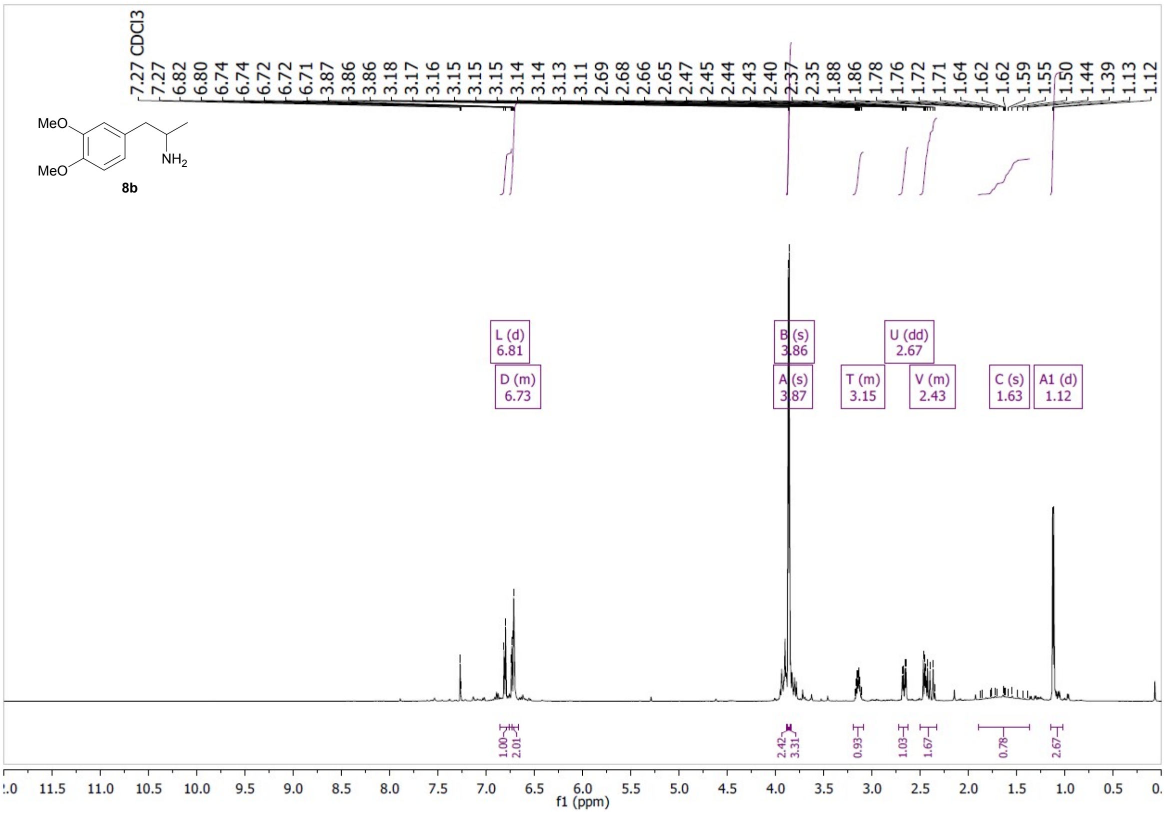

Figure S28. ^{1}H-NMR spectrum of 1-(3,4-dimethoxyphenyl)propan-2-amine (**8b**)

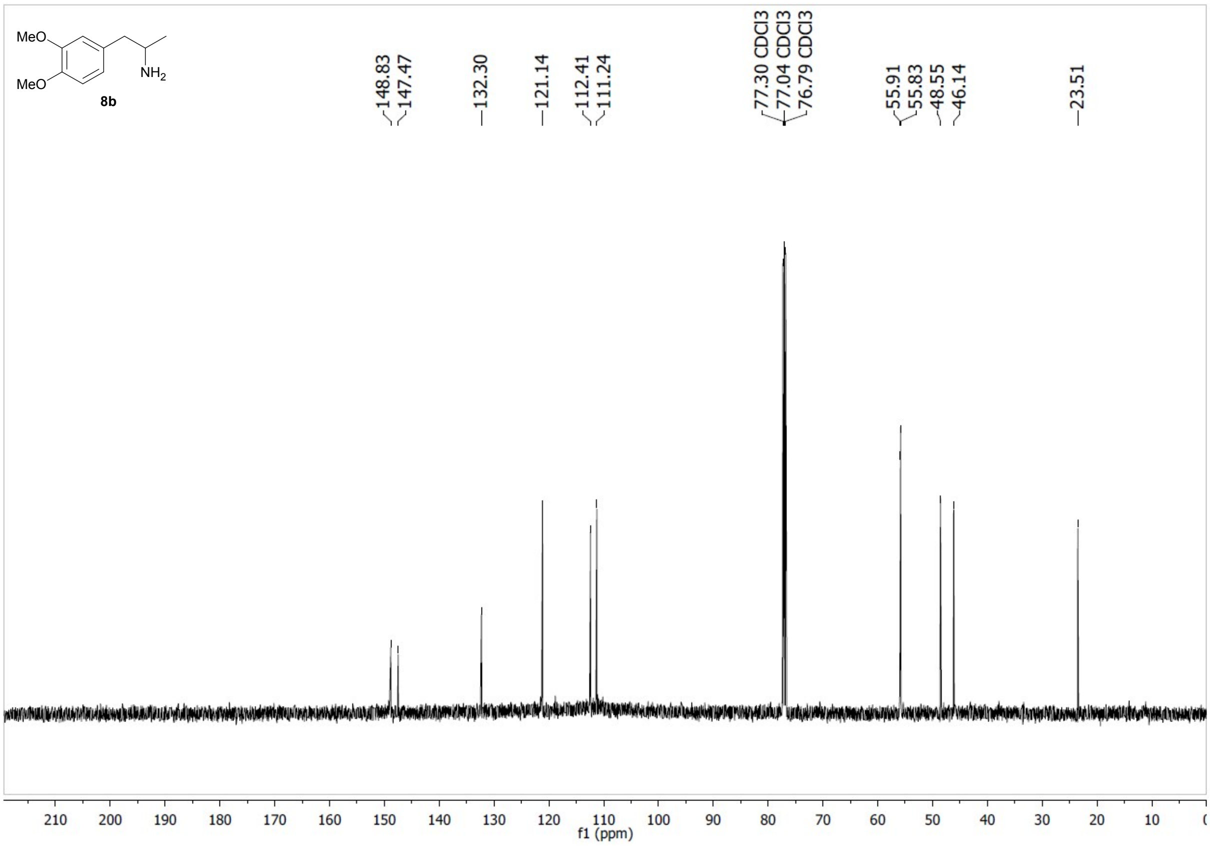

Figure S29. ^{13}C-NMR spectrum of 1-(3,4-dimethoxyphenyl)propan-2-amine (**8b**)

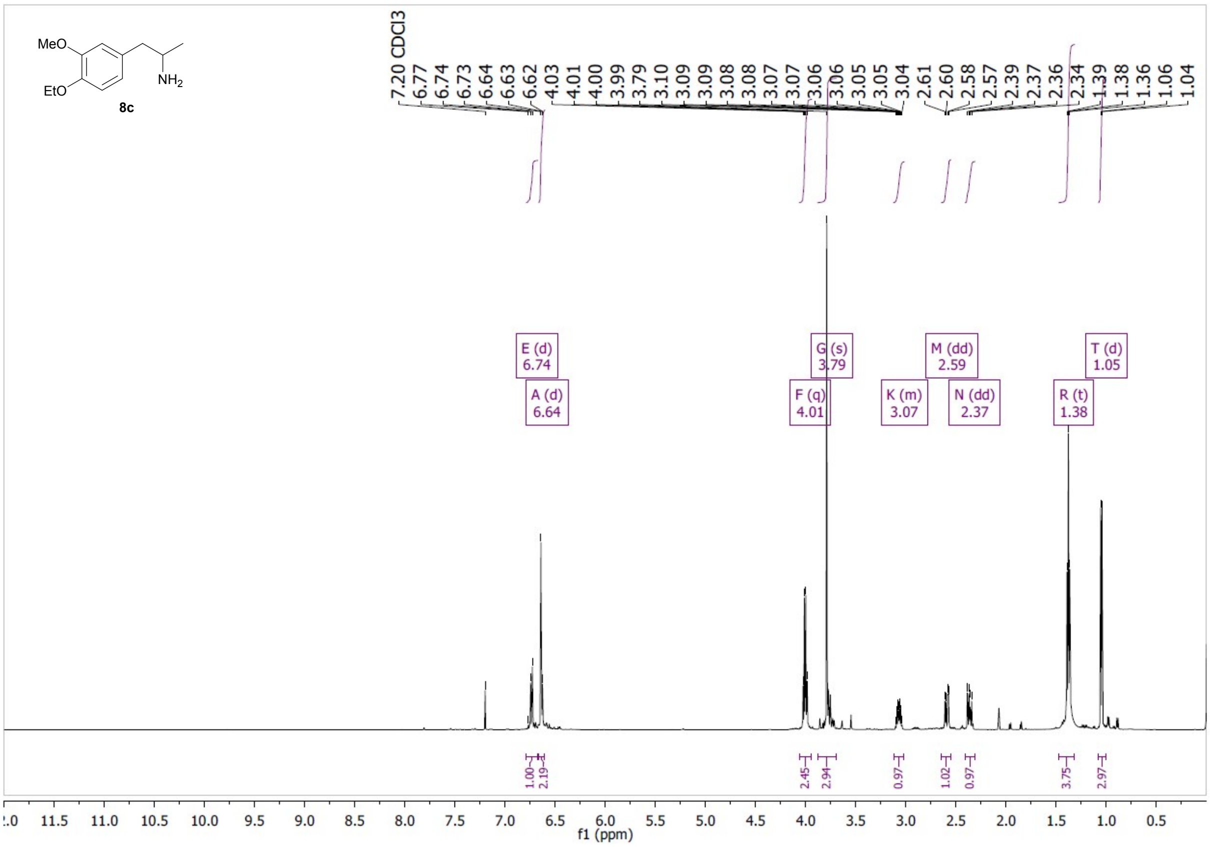

Figure S30. ^{1}H-NMR spectrum of 1-(4-ethoxy-3-methoxyphenyl)propan-2-amine (**8c**)

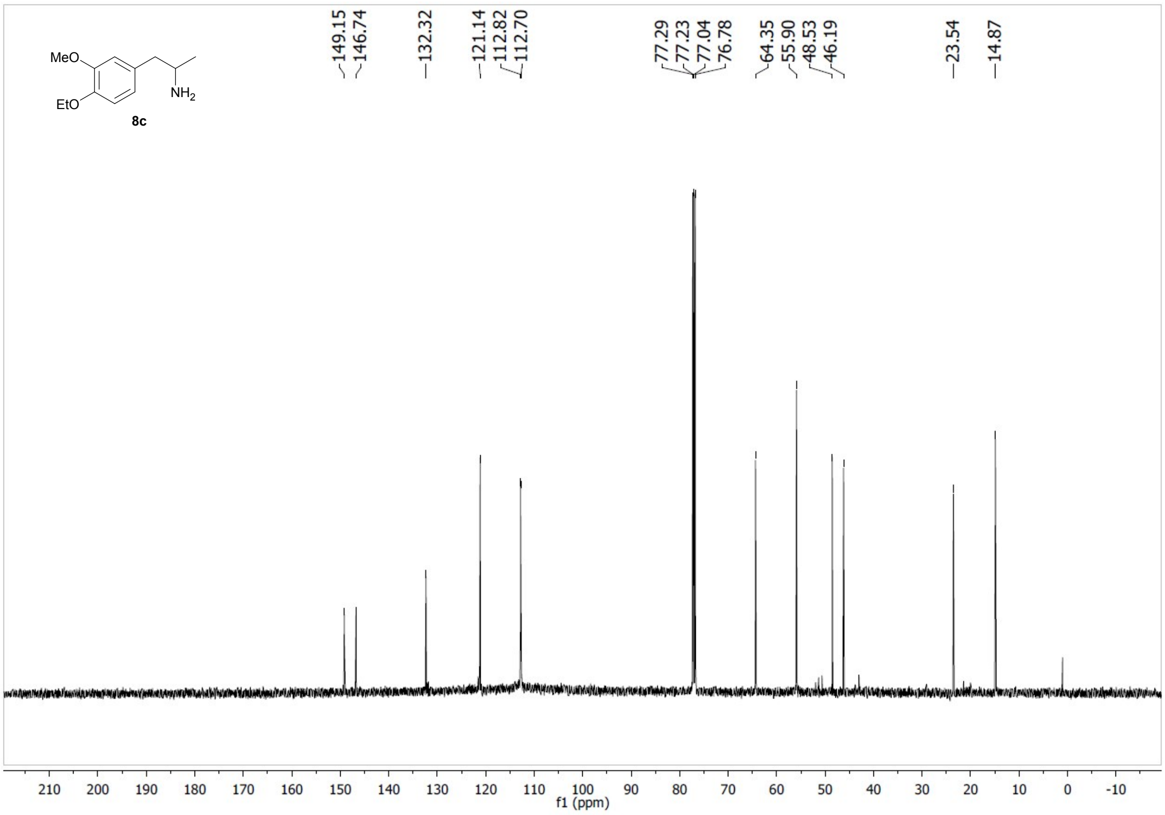

Figure S31. ^{13}C-NMR spectrum of 1-(4-ethoxy-3-methoxyphenyl)propan-2-amine (**8c**)

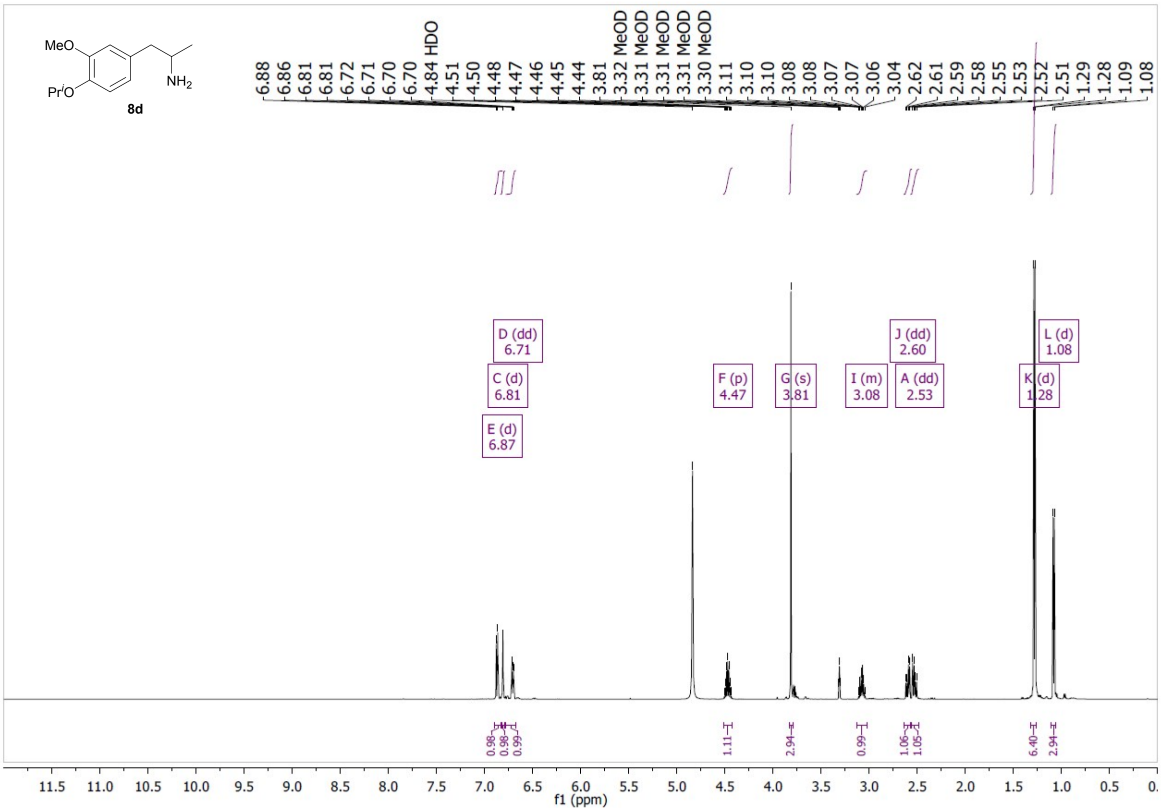

Figure S32. ^{1}H-NMR spectrum of 1-(4-isopropoxy-3-methoxyphenyl)propan-2-amine (**8d**)

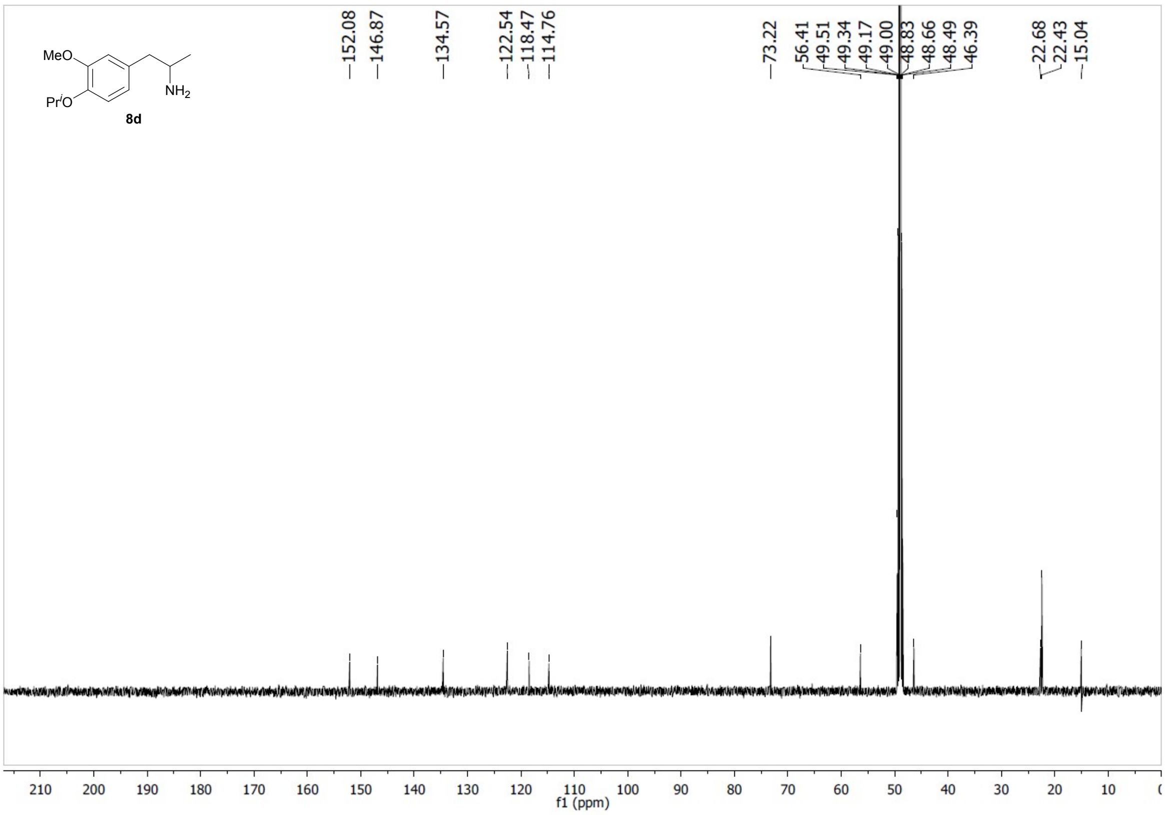

Figure S33. ^{13}C-NMR spectrum of 1-(4-isopropoxy-3-methoxyphenyl)propan-2-amine (**8d**)
